# Repeat-Induced Point Mutation and Gene Conversion Coinciding with Heterochromatin Shape the Genome of a Plant-Pathogenic Fungus

**DOI:** 10.1128/mbio.03290-22

**Published:** 2023-04-24

**Authors:** Jovan Komluski, Michael Habig, Eva H. Stukenbrock

**Affiliations:** a Environmental Genomics, Christian-Albrechts University of Kiel, Kiel, Germany; b Max Planck Institute for Evolutionary Biology, Plön, Germany; University of Georgia; University of California, Berkeley

**Keywords:** repeat-induced point mutation (RIP), meiotic mutation, tetrad analysis, meiosis, gene conversion, epigenetics

## Abstract

Meiosis is associated with genetic changes in the genome—via recombination, gene conversion, and mutations. The occurrence of gene conversion and mutations during meiosis may further be influenced by the chromatin conformation, similar to the effect of the chromatin conformation on the mitotic mutation rate. To date, however, the exact distribution and type of meiosis-associated changes and the role of the chromatin conformation in this context are largely unexplored. Here, we determine recombination, gene conversion, and *de novo* mutations using whole-genome sequencing of all meiotic products of 23 individual meioses in Zymoseptoria tritici, an important pathogen of wheat. We confirm a high genome-wide recombination rate of 65 centimorgan (cM)/Mb and see higher recombination rates on the accessory compared to core chromosomes. A substantial fraction of 0.16% of all polymorphic markers was affected by gene conversions, showing a weak GC-bias and occurring at higher frequency in regions of constitutive heterochromatin, indicated by the histone modification H3K9me3. The *de novo* mutation rate associated with meiosis was approximately three orders of magnitude higher than the corresponding mitotic mutation rate. Importantly, repeat-induced point mutation (RIP), a fungal defense mechanism against duplicated sequences, is active in *Z. tritici* and responsible for the majority of these *de novo* meiotic mutations. Our results indicate that the genetic changes associated with meiosis are a major source of variability in the genome of an important plant pathogen and shape its evolutionary trajectory.

## INTRODUCTION

Meiosis is an important mechanism of genome evolution, as it generates genetic variability for selection to act upon. Since all changes to the genome that occur prior to and during meiosis are potentially affecting the germline, meiosis is a pivotal mechanism in shaping the evolutionary trajectory of sexually propagating species. Three major classes of genetic changes are associated with meiosis: (i) recombination, (ii) gene conversion, and (iii) meiotic mutations.

Recombination is the reciprocal exchange of information between homologous chromosomes during meiosis (1). Canonical meiosis is initiated by the formation of double-strand breaks (DSBs) by the topoisomerase-like protein Spo11 at many genomic locations (2, 3). Indeed, these DSBs, which are resected to generate single-stranded DNA overhangs that can invade the homologous chromosome, are thought to guide chromosome pairing in many species (3, 4). Most DSBs are repaired and resolved as non-crossover (NCO) events which on some occasions are associated with gene conversions, while few DSBs will be resolved to crossover (CO) events and the reciprocal exchange of larger chromosome portions between homologous chromosomes (4–8). Interestingly, recombination rates, i.e., the rate of reciprocal exchanges of chromosome sections during meiosis, vary considerably between species (9, 10). The highest CO frequencies reported so far are in fission yeast, where an average of eleven to nine COs per chromosome pair by far exceed the minimum of one crossover event per chromosome considered to be required for proper chromosome pairing and segregation—with the average number of COs per chromosome in most species rarely exceeding three (11–14). Recombination rates also vary along chromosomes, with crossovers occurring in hotspots (15–18) and being mostly absent in centromeric regions (14, 19). Where crossovers are occurring seems to be affected by the synaptonemal complex, a protein structure that forms along meiotic chromosomes, as well as the chromatin structure that appears to influence the location of the DSBs (9). Accessible chromatin appears to be a hotspot for DSBs, as shown in Saccharomyces cerevisiae, where DSBs are primarily located within regions of accessible chromatin generally found at gene promoters (20). Generally, heterochromatic marks are associated with lower recombination rates, while euchromatic marks are associated with elevated recombination rates (3, 9, 21–24). During the last decade, the use of population data for the determination of recombination rates became feasible based on the rapidly increasing availability of whole-genome sequencing data (17, 18, 25, 26). However, progeny analysis and tetrad analysis are still required to analyze all the genetic processes associated with crossover events.

Gene conversion is also one of the possible outcomes of DSB formation and resolution during meiosis, but in contrast to recombination, gene conversion directly affects the allele frequency. Gene conversion describes the non-reciprocal (i.e., unidirectional) transfer of a sequence from one locus (the donor) to a different genetic locus (the acceptor) (27). Gene conversions can either be interallelic or nonallelic (also called interlocus). The first will result in changes in the allele frequency, while the latter is (next to unequal crossovers) involved in gene duplication, gene expansion, and homogenization of gene families and has, for example, been observed in gene families involved in host-pathogen interactions (27–30). Gene conversion is initiated by DSBs, followed by resection of the DSB end and the invasion of the single-stranded tail into homologous sequences. Sequence differences between the two homologous sequences will result in partially mismatched heteroduplex DNA (5, 27, 31). If the mismatch is repaired using the information of the invading DNA, the acceptor allele will be changed to the donor allele and hence result in gene conversion as manifested by a 3:1 rather than 2:2 segregation pattern in the resulting products of a single meiosis—a tetrad. Heteroduplex DNA and repair also occur during crossover (CO) events, and hence, gene conversions can be categorized into those associated with CO (CO-GC) and those associated with non-crossover NCO (NCO-GC) (31). Rates of gene conversion vary considerably between species (32, 33), which appears to be mainly influenced by the tract length (i.e., the length of the sequence containing converted markers) and recombination rates (5, 33, 34). Gene conversion in some species seems to be GC-biased, probably caused by the GC-biased repair of A:C and G:T in the heteroduplex DNA (35, 36), which is assumed to have important consequences on the equilibrium GC content of the genomes (33, 37). Biased gene conversion may, however, not be universally important, as it was found not to occur in some fungi as well as in some plant and algae species (18). Although chromatin configuration and hence the histone modifications are assumed to affect the rate of gene conversions, such an association was so far not identified.

Finally, meiosis is also associated with mutations that occur before or during meiosis (38–40). Since mutations on average are considered to be deleterious, mutation rates, in general, are low but can also differ greatly between species (41). Meiosis-associated mutation rates, in turn, can differ greatly from the corresponding mitotic mutation rate in the same species, indicating different mechanisms and/or constraints. For example, the germline mutation rate in humans and mice is 1.2 × 10^−8^ or 5.7 × 10^−9^ per nucleotide per generation, respectively, two orders of magnitude lower than the corresponding mitotic mutation rate (10, 42, 43). Here, germline mutations might be linked to DSBs and their repair (44), with a higher number of mutations occurring in the vicinity of recombination events (45). Estimates of meiosis-associated mutation rates in different fungal species also vary considerably. In S. cerevisiae, the meiosis-associated mutation rate is 8 × 10^−8^ per bp per cell generation (44), much higher than the mitotic mutation rate of 3.3 × 10^−10^ per bp per cell division (46). In Neurospora crassa, the meiosis-associated mutation rate is very high at 3.38 × 10^−6^ per bp per generation (47), contrasting with a much lower mitotic mutation rate of 6.7 × 10^−10^ per bp per cell division (48). Interestingly, this extremely high meiotic mutation rate in N. crassa is caused by repeat-induced point mutation (RIP), a fungal defense mechanism against duplicated sequences (Fig. S1A) (40, 49). RIP is restricted to haploid parental nuclei just prior to karyogamy and meiosis and acts on duplicated sequences of a minimum length of 400 bp in N. crassa (50–52). Once recognized, both duplicated sequences will be mutated in a C→T manner (50–52), and RIP can sometimes leak into adjacent non-repetitive regions (53, 54). RIP signatures have been detected in the genomic sequences of many fungi; however, active RIP was experimentally confirmed in only a few fungal species (47, 55). Hence, there is a growing body of evidence that suggests that the mutational processes prior to and during meiosis differ from those during mitosis. In fungi, the meiotic mutation rate appears to be higher than the mitotic mutation rate, which for some fungi is assumed to be the result of RIP.

10.1128/mbio.03290-22.5 FIG S1 Distribution of SNP densities, recombination rates, and the number of crossovers (CO) per chromosome. (A) Schematic overview of the assumed sexual process in *Z. tritici* (114, 115). The spermatial nucleus is transferred via the trichogyne to the ascogonium, resulting in plasmogamy and a dikaryon with two separate nuclei. During the dikaryon stage, RIP is assumed to occur, affecting replicated regions (cyan) and introducing C→T lesions that can be resolved either early, before premeiotic DNA replication, resulting in a 2:2 segregation pattern (red background), or late, with premeiotic DNA replication resolving the lesions, resulting in a 1:3 segregation pattern (green background). Meiosis I segregates homologous chromosomes, followed by chromatid separation in meiosis II. A subsequent mitosis results in the production of eight ascospores contained in an ascus (not shown). (B) SNP density in SNPs per 1,000 bp comparing the two parental strains, IPO323 and IPO94269. (C) Distribution of distances between two neighboring SNPs. (D) Recombination rates per chromosome. Note that the number of crossovers on the accessory chromosomes showed little variation between tetrads (e.g., 0 to 3 for chromosome 14); thus, the estimated recombination rate also exhibited little variation. Box plots display the following: center line, median; box limits, upper and lower quartiles; whiskers, 1.5× interquartile range; points, crossover frequency per genomic compartment per tetrad. (E) Average number of COs per chromosome. Points in panel E depict the average CO number for the respective chromosome length; blue points represent core chromosomes; orange points represent accessory chromosomes; the dashed line shows Pearson’s correlation. Download FIG S1, TIF file, 2.5 MB. Copyright © 2023 Komluski et al. 2023 Komluski et al. https://creativecommons.org/licenses/by/4.0/ This content is distributed under the terms of the Creative Commons Attribution 4.0 International license.

Here, we use tetrad analysis to determine genetic changes associated with meiosis in the ascomycete fungus Zymoseptoria tritici, a pathogen of wheat. The haploid genome of *Z. tritici* comprises 13 core chromosomes and a set of smaller accessory chromosomes (56). These non-essential accessory chromosomes carry a fitness cost (57), are enriched in the facultative heterochromatin mark H3K27me3 (58), have a higher mutation rate during mitosis (59), and exhibit a meiotic drive (60). The availability of complete tetrads for *Z. tritici* allows us here to address all three major classes of genetic changes associated with sexual reproduction. In particular, the frequency and distribution of mutations associated with sexual reproduction are unknown, although *Z. tritici* has an asexual and a sexual reproductive cycle, with the latter being the main source of the primary inoculum during the initial stages of the infection (61). The mitotic mutation rate in *Z. tritici* has been determined experimentally by mutation accumulation experiments at 3.2 × 10^−10^ per bp per mitotic cell division (59), which is similar in other fungi (46, 48). Although histone modifications affect the mitotic mutation rate of *Z. tritici* (59, 62), it is unknown if the distribution of meiotic recombination events, gene conversion, and meiosis-associated mutations are also influenced by these histone modifications. Finally, although the genome of *Z. tritici* shows signatures of RIP (56), RIP has so far not been demonstrated experimentally, and the efficacy of this mutational mechanism in this pathogen is not known. Given the fact that 18.6% of the genome of *Z. tritici* is represented by transposable elements (TEs), it is plausible that RIP is less efficient in *Z. tritici* than in N. crassa or fails to recognize some duplicated regions (63). Here, we study all major classes of meiosis-associated genetic changes in *Z. tritici* by analyzing whole-genome sequences of complete tetrads to (i) estimate recombination and gene conversion rates for core and accessory chromosomes, (ii) determine the association between recombination and gene conversions with chromatin modifications, and (iii) estimate meiotic mutation rates in *Z. tritici*. The use of tetrads allowed us to detect and describe the effects of active RIP and generate a fine-scale map of recombination and gene conversion events and its association with chromatin modifications.

## RESULTS

### Accessory chromosomes show higher recombination rates.

**FIG 1:**
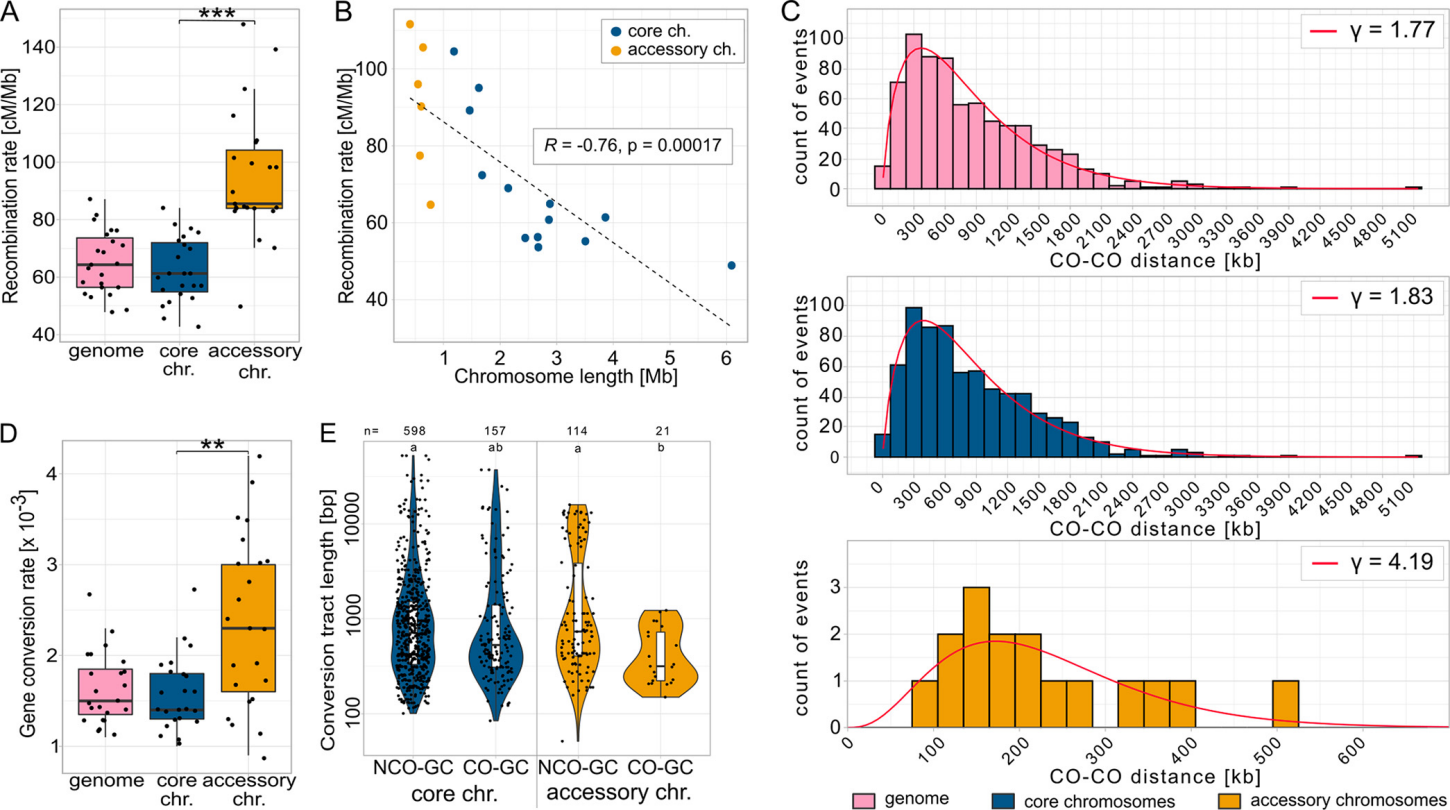
Comparison of recombination rates, gene conversion rates, and crossover interference for various genomic compartments. (A) Recombination rates for the genome and on core and accessory chromosomes. (B) Correlation between recombination rates and chromosome length. (C) Crossover (CO) interference. Distribution of distances between adjacent CO events (CO-CO distance) in the entire genome (pink), on core chromosomes (blue), and on accessory chromosomes (yellow). The red line represents the fitted gamma distribution for the observed CO-CO distances. γ values higher than 1 indicate positive CO interference, i.e., a lower than expected number of small CO-CO distances. (D) Gene conversion rates for the genome and core and accessory chromosomes. (E) Violin plot of non-crossover-associated gene conversion (NCO-GCs) and crossover-associated gene conversion (CO-GCs) tract lengths for core and accessory chromosomes. The tract lengths of the gene conversion events spanning TEs are excluded (different letters depict significantly different groups with a *P* value of <0.05 in the Wilcoxon test with Bonferroni correction). In panels A and D, *P* values of paired Wilcoxon tests are shown (*, *P* < 0.05; **, *P* < 0.005; ***, *P* < 0.0005).

**FIG 2:**
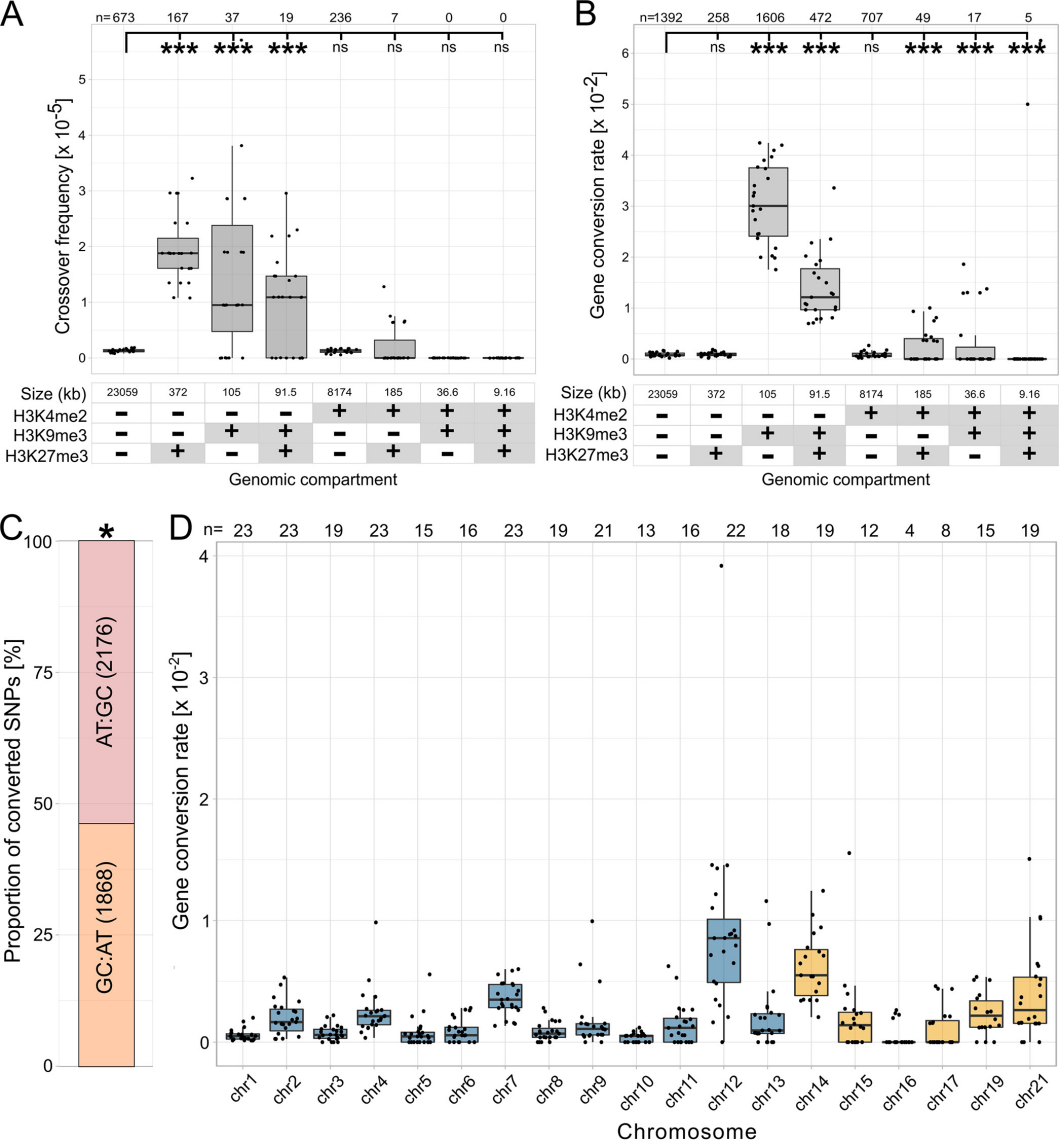
Correlation between crossover frequency and gene conversion rates with histone modifications, GC-biased gene conversion, and gene conversion rate per chromosome. (A and B) Correlation between (A) crossover frequencies or (B) gene conversion rates and chromatin modifications. The gene conversion rate is calculated as the number of converted markers per genomic compartment divided by the total number of markers in the genomic compartment. A genomic compartment is defined by all regions of the genome that share the presence/absence of the indicated histone modifications. The presence/absence of the specific chromatin modification (H3K4me2, H3K9me3, or H3K27me3, respectively) in the genomic compartment is depicted with + or – in the table below the *x* axis. χ^2^-test *P* values for comparing the indicated genomic compartments with the genomic compartment lacking all the indicated histone modifications are shown (*, *P* < 0.05; **, *P* < 0.005; ***, *P* < 0.0005; ns, not significant). (C) GC-biased gene conversion in *Z. tritici*. The stacked barplot shows the proportion of AT to GC converted markers and the proportion of GC to AT converted markers. Binomial test *P* values are shown (*, *P < *0.05; **, *P < *0.005; ***, *P < *0.0005). (D) Gene conversion rate per chromosome calculated as the proportion of converted markers from the total number of markers on the respective chromosome. The numbers above box plots show the number of tetrads with gene conversion detected on the respective chromosome. Box plots display center line, median; box limits, upper and lower quartiles; whiskers, 1.5× interquartile range; points, rate per tetrad.

To determine the distribution of recombination events during meiosis in *Z. tritici*, we used previously published tetrads and obtained whole-genome sequences for the tetrad progenies (60). To this end, we included 23 tetrads comprising four ascospore isolates, totaling 92 genomes. An average of 118,772 single nucleotide polymorphisms (SNPs; 0.3% of all analyzed genomic sites) per tetrad was used for the analysis (see Materials and Methods), with the SNP density being similar between core and accessory chromosomes (Fig. S1B). The median distance between SNPs was 61 bp, with few instances of distances between SNPs exceeding 5,000 bp (Fig. S1C). From these data, we identified individual recombination events with the CrossOver tool from the ReCombine package (64) and calculated the recombination rate. A total of 1,138 crossover events were observed, resulting in a genome-wide recombination rate of 65 centimorgan (cM)/Mb, consistent with previously published estimates of the recombination rate in this fungus. Intriguingly, the recombination rate was significantly higher on accessory chromosomes (92.7 cM/Mb) than on the core chromosomes (62.6 cM/Mb) (Fig. 1A, Fig. S1D, Table S1A and B). The recombination rate was negatively correlated with the chromosome length (Pearson’s *R* = –0.76, *P* = 0.00017) (Fig. 1B), but the absolute number of crossovers per chromosome was positively correlated with the chromosome length (Pearson’s *R* = 0.96, *P* = 6.2 × 10^−11^) (Fig. S1E). When two or more crossovers are present in a bivalent, they tend not to occur near each other in many species—a process called crossover interference (65). We accessed the distribution of the distances between COs using the gamma distribution (66–68) and detected crossover interference (γ > 1) for both core and accessory chromosomes (see Fig. 1C). When correlating the crossover frequencies with regions enriched in heterochromatin marks, we found that regions enriched in heterochromatin marks (H3K27me3 or H3K9me3) were associated with higher crossover frequencies, whereas the euchromatin mark H3K4me2 did not show a higher recombination rate than regions lacking all three marks (Fig. 2A).

10.1128/mbio.03290-22.2 TABLE S1 **A** List of all crossover and associated gene conversions **B** Summary of crossover frequencies per tetrad for the core and accessory chromosomes **C** List of genes in crossover hotspots. Download Table S1, XLSX file, 0.1 MB. Copyright © 2023 Komluski et al. 2023 Komluski et al. https://creativecommons.org/licenses/by/4.0/ This content is distributed under the terms of the Creative Commons Attribution 4.0 International license.

To further assess the variation in recombination rate across the genome, we estimated the recombination rates in 20 kb non-overlapping windows. We defined hotspots as those 20 kb windows that showed a significantly (*P* < 0.001) higher number of recombination events than expected as determined by the Poisson distribution and identified a total of 52 recombination hotspots with more than 4 crossover events per 20 kb window. Recombination rates per window were highly variable, ranging from 0 to 1,196 cM/Mb (Fig. S2). We conclude that recombination varies considerably across the genome, occurs in hotspots, and is higher on the accessory than on the core chromosomes. A more detailed analysis of the immediate vicinity of the crossover events allowed us to identify four motifs that were significantly overrepresented in the 1 kb sequences surrounding the crossover and to correlate recombination rates with short sequence repeats (SSRs) (Fig. S3C and D). However, a more fine-scale analysis of the nine 1 kb windows with ≥4 crossover events showed no apparent correlation between the location of TEs, SSRs, genes, and the three histone modifications, H3K4me2, H3K9me3, and H3K27me3 (Fig. S3A). We considered the relevance of high recombination rates with regard to gene evolution and correlated the recombination map with the coordinates of protein-coding genes. In total, we observed 376 genes in the CO hotspots. From the 376 genes observed in the CO hotspots, 6 encode effector candidates and 9 encode CAZymes (Table S1C), which are gene categories with a putative relevance in the pathogenicity of the fungus.

10.1128/mbio.03290-22.6 FIG S2 Distribution of recombination rates along the chromosomes in *Z. tritici*. (A) Distribution of recombination rates on core chromosomes. (B) Distribution of recombination rates on accessory chromosomes. Chromosomes are divided in 20 kb non-overlapping windows. The horizontal dashed pink line represent the threshold for hotspots with *P* < 0.001 defined by the Poisson distribution (more than four crossovers per 20 kb window). The blue lines represent the crossover distribution on core chromosomes, orange lines represent the crossover distribution on accessory chromosomes, and red horizontal lines show the centromere position. The *x* axis designates the chromosome length. The black rectangles on the *x* axis depict transposable elements. (C) Number of hotspots on the indicated chromosomes. Download FIG S2, TIF file, 1.8 MB. Copyright © 2023 Komluski et al. 2023 Komluski et al. https://creativecommons.org/licenses/by/4.0/ This content is distributed under the terms of the Creative Commons Attribution 4.0 International license.

10.1128/mbio.03290-22.7 FIG S3 Details of the location of crossover events. (A and B) Display of the genomic features that are associated with the nine 1 kb windows that contained the highest number of crossover events (≥4) for (A) core chromosomes and (B) accessory chromosomes. (C) The four most significantly overrepresented short motifs (*P* < 1 × 10^−15^) in the vicinity of crossover events (500 bp upstream and downstream). (D) Recombination rate comparison between regions that are covered by simple sequence repeats (SSR; size, 39 kb) and the rest of the genome. Statistical significance as determined by Fisher’s exact test is depicted (*, *P* = 0.031). Download FIG S3, TIF file, 1.4 MB. Copyright © 2023 Komluski et al. 2023 Komluski et al. https://creativecommons.org/licenses/by/4.0/ This content is distributed under the terms of the Creative Commons Attribution 4.0 International license.

### High gene conversion rates are more uniformly distributed within the genome.

The genomes of ascospore progenies resulting from one meiotic event provided us with a unique opportunity to characterize the location and distribution of gene conversion events. We therefore identified gene conversion events along the genome and compared the gene conversion rate for different genomic compartments. We identified a total of 890 gene conversion events, 712 associated with non-crossover events (non-crossover gene conversion [NCO-GC], 80%) and 178 associated with crossover events (crossover gene conversion [CO-GC], 20%) (Table S2A). Each of these gene conversion events was associated with at least three converted markers (SNPs), with the total number of converted markers being 3,529 and 979 for NCO-GC and CO-GC, respectively. We distinguished the gene conversion events on core and accessory chromosomes of *Z. tritici* and found that 755 gene conversion events were located on core and 135 on accessory chromosomes (Table S2B). We next explored the general patterns of converted SNPs. We observed a weak GC-bias in the gene conversions (binomial test, *P* = 0.0215; Fig. 2C). Based on genome-wide SNPs identified among the ascospore isolates, we found that the genome-wide gene conversion rate was 1.6 × 10^−3^ per SNP (Fig. 1D, Table S2B). The gene conversion rate was significantly higher on the accessory chromosomes than on the core chromosomes (paired Wilcoxon signed rank test, *P* = 1.0 × 10^−3^) (Fig. 1D, Fig. 2D). Tract length is considered to be one of the main determinants of gene conversion rates; therefore, we estimated the median conversion tract length for both types of gene conversion events. The median tract length for non-crossover (NCO-GC) was 539 bp, and 432 bp for gene conversions associated with crossovers (CO-GC), respectively (Fig. 1E). Although the tract length on the accessory chromosomes appeared to differ from the corresponding tract length on the core chromosomes, none of these comparisons were significant (e.g., NCO-GC accessory versus core chromosomes *P* = 0.927) (Fig. 1E).

10.1128/mbio.03290-22.3 TABLE S2 **A** List of all identified gene conversions **B** Summary of the number of SNPs and converted SNPs (total, core [= on core chromosomes], accessory [= on accessory chromosomes]) **C** List of genes in gene conversion hotspots **D** Summary of the converted marker and non-converted marker in genomic compartments with the indicated presence/absence of specific histone modifications. Download Table S2, XLSX file, 0.1 MB. Copyright © 2023 Komluski et al. 2023 Komluski et al. https://creativecommons.org/licenses/by/4.0/ This content is distributed under the terms of the Creative Commons Attribution 4.0 International license.

In the same way as we characterized the distribution of recombination hotspots along the fungal genomes, we used the map of gene conversion events to identify regions with exceptionally high gene conversion rates, here defined as gene conversion hotspots. First, to assess the distribution of gene conversion, we divided the genome into 20 kb non-overlapping windows and calculated the number of gene conversion events in each window. We identified 32 gene conversion hotspots with more than 4 gene conversion events per 20 kb window that were significantly different from the background with *P* value of < 0.001 defined by the Poisson distribution (Fig. S4A to C). Of the 145 genes in the gene conversion hotspots, 2 genes encode for effector candidates and seven genes encode for CAZymes (Table S2C). We speculate that the increased rate of gene conversion in these putative pathogenicity-related genes could be a putative mechanism of rapid evolution. However, the majority of the genes associated with gene conversion hotspots (95 genes) encode proteins that have not yet been functionally annotated, preventing any meaningful enrichment analysis. Taken together, the ascospore population allowed us to precisely map and characterize gene conversion events in *Z. tritici*. We found that rates of gene conversion showed less variation across the genome than recombination rates, but with both being higher on accessory chromosomes than on core chromosomes.

10.1128/mbio.03290-22.8 FIG S4 Distribution of gene conversions along the chromosomes in *Z. tritici*. (A) Distribution of gene conversions on core chromosomes. (B) Distribution of gene conversions on accessory chromosomes. Chromosomes are divided into 20 kb non-overlapping windows. The horizontal dashed pink line represents the threshold for hotspots with *P* < 0.001 defined by Poisson distribution (more than 4 gene conversion events per 20 kb window). The blue lines represent crossover distribution on core chromosomes, orange lines represent crossover distribution on accessory chromosomes, and red horizontal lines show the centromere position. The *x* axis designates the chromosome length. Black rectangles on *x* axis depict transposable elements. The *y* axis shows the number of gene conversion events (NCO-GC and CO-GC). (C) Number of gene conversion hotspots on the indicated chromosomes. Download FIG S4, TIF file, 1.3 MB. Copyright © 2023 Komluski et al. 2023 Komluski et al. https://creativecommons.org/licenses/by/4.0/ This content is distributed under the terms of the Creative Commons Attribution 4.0 International license.

### Gene conversion rates are higher in regions enriched in heterochromatin modifications.

Previous studies in chicken B-cell lines have identified a correlation of chromatin structure with gene conversion (69), but detailed analysis of the effect of specific histone modifications is mostly missing. Thus, to investigate the potential effect of histone modifications on the rate of gene conversion in *Z. tritici*, we conducted a correlation analysis of maps of histone modifications and gene conversion rates. We focused on three histone marks which have been previously well characterized in *Z. tritici* using chromatin immunoprecipitation of antibodies targeting specific histone modifications followed by sequencing (ChIP-seq): the euchromatin mark H3K4me2, the constitutive heterochromatin mark H3K9me3, and the facultative heterochromatin mark H3K27me3 (58) (Fig. 2C, Table S2D). Importantly, we excluded TEs from the full-factorial analysis of gene conversions, and therefore, regions enriched with H3K9me3 and H3K27me3 in this analysis are not associated with TEs. Our analyses showed that higher gene conversion rates associate with regions enriched in heterochromatin marks, particularly in regions solely enriched with H3K9me3, as well as in regions enriched with both H3K9me3 and H3K27me3 (Fig. 2B). Regions enriched solely in H3K27me3 were not associated with higher gene conversion rates, which contrasts with their correlation with the highest crossover frequencies (Fig. 2A). The median gene conversion rate in the regions enriched with H3K9me3 was 3 × 10^−2^ per converted marker per tetrad per meiosis, and the median gene conversion rate in the H3K9me3 and H3K27me3 regions was 1.3 × 10^−2^ per converted marker per tetrad per meiosis. Genome regions not enriched with either of the three histone marks, H3K4me2, H3K9me3, and H3K27me3, showed the lowest gene conversion rate of 0.9 × 10^−3^. In summary, the histone maps allowed us to reveal that repressive heterochromatin modifications, especially H3K9me3 but not H3K27me3, are associated with higher gene conversion rates in *Z. tritici*.

### The majority of meiotic mutations are caused by RIP.

**FIG 3:**
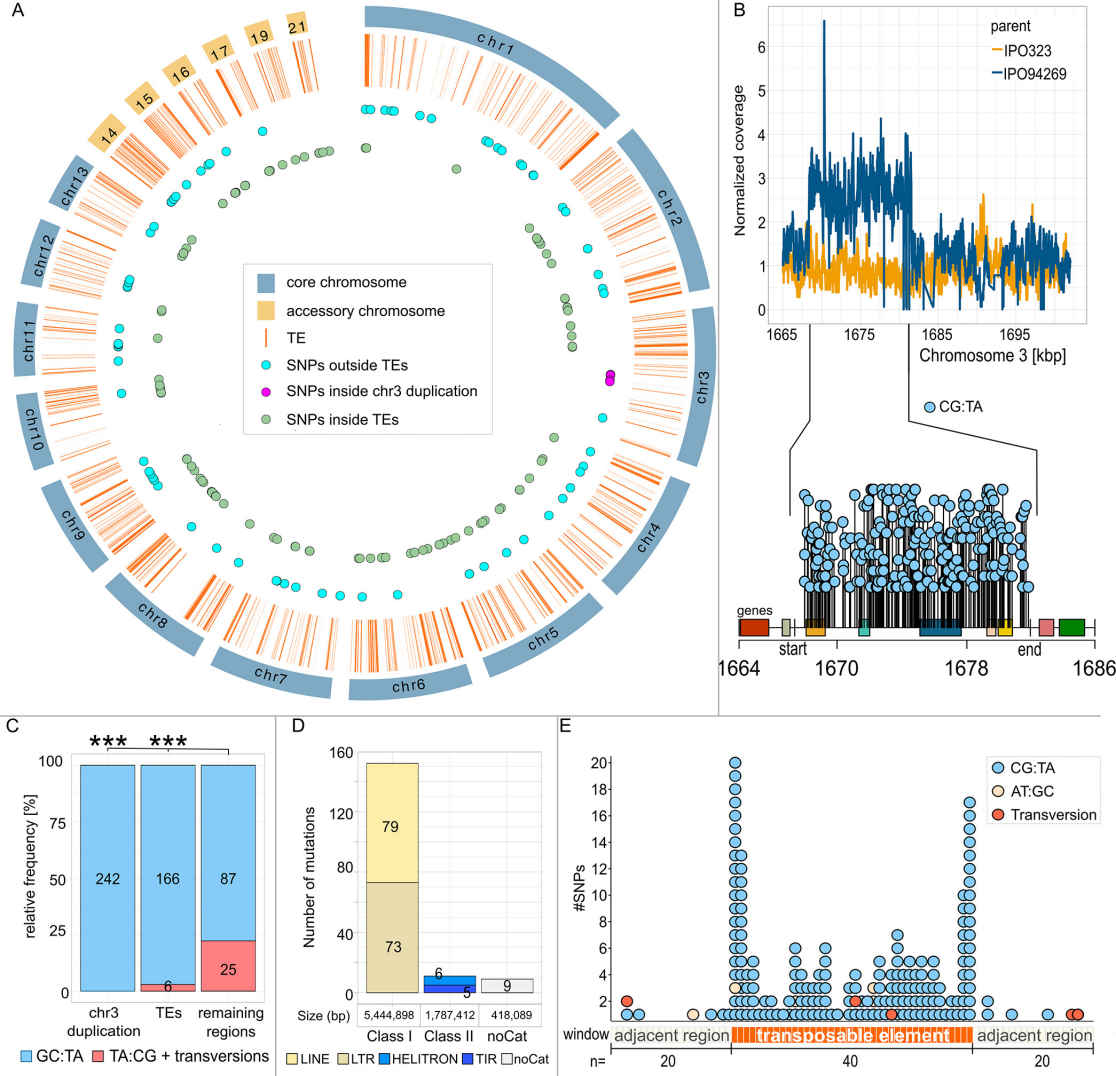
Genome-wide distribution of *de novo* mutations associated with meiosis in *Z. tritici*. (A) CircosPlot of the meiotic SNP distribution in the genomic features of *Z. tritici* (orange lines, TEs; blue dots, meiotic SNPs outside the TEs; lilac dots, meiotic SNPs inside the duplication on chromosome 3; green dots, meiotic SNPs inside the TEs). (B) RIP-like mutations in the duplicated 14 kb region on chromosome 3. The top line graph shows the difference in normalized coverage between IPO323 and the IPO94269 parent in the region on chromosome 3. The distributions of mutations in the duplicated chromosome 3 region and 10 kb upstream and downstream regions are shown in the lolliplot below the line graph. The start and the end on the *x* axis of the lolliplot designate the start and the end of the duplicated region. Rectangles in different colors depict the genes located in this region. Each lollipop represents a single mutation. (C) Number of meiotic mutations in and out of the RIP active regions represented as their relative frequencies. CG:TA transitions are colored in light blue, and TA:CG transitions and transversions are colored in red. Fisher exact test *P* values are shown (*, *P* < 0.05; **, *P* < 0.005; ***, *P* < 0.0005). (D) Number of *de novo* mutations in different classes and families of repeats (light yellow, long interspersed nuclear element [LINE]; dark yellow, long terminal repeats [LTR]; marine blue, HELITRON; dark blue, tandem inverted repeats [TIR]; white, noCat). (E) Distribution of meiotic SNPs along TEs. Each TE was divided into 40 equal-sized windows. Each black rectangle on the *x* axis represents a window inside a TE representing 2.5% of the TE length. Beige rectangles represent windows in the regions directly adjacent to TEs. Dots above rectangles represent one mutation in each window (yellow dots, AT:GC transitions; blue dots, CG:TA transitions, red dots, transversions).

Recombination and gene conversion as well as meiosis and meiosis-associated processes themselves are considered to be mutagenic (38–40, 45). Hence, we asked to what extent *de novo* mutations had occurred in the ascospore progenies. These mutations are distinguished by being absent in both of the parental strains but present in the ascospores of the tetrad. Meiotic mutation rates often differ from mitotic mutation rates and are associated with recombination or premeiotic processes (39, 42–46). We observed a total of 526 *de novo* mutations that were absent in both of the parental strains for the ascospores of the 23 tetrads. Hence, through the sexual cycle, there were, on average, 22.9 mutations per genome per generation, resulting in a mutation rate of 5.7 × 10^−7^ mutations per bp per generation (Fig. 3A, Table S3A). Of these mutations that originated from the sexual cycle, 242 (42%) resided in one particular region that clearly stood out as we mapped meiotic SNPs along the genome. This high number of SNPs were located in a 14,001 bp region on chromosome 3 (Fig. 3A to C). Each of the 242 mutations in this region on chromosome 3 was a CG:TA transition. As we further inspected this region, we noted that the 14 kb region spanning 1,668,370 bp to 1,682,371 bp on chromosome 3 showed an increased sequencing coverage corresponding to a duplication of the region in the parent IPO94269 (Fig. 3B). This means that this duplication on chromosome 3 was present in all 23 meioses and could possibly be identified by RIP, a genome defense mechanism against such duplicated regions (40). A high number of transitions in a duplicated region is the hallmark of such active RIP. Indeed, the high number of mutations in the duplicated region and the fact that all of these mutations are CG:TA transitions indicates active RIP in *Z. tritici*. Interestingly, the duplicated region on chromosome 3 showed RIP mutations in only 10 of the 23 tetrads (Fig. S5A). Further examination of the sequencing coverage depth revealed that, despite being present in the parental strain IPO94269, the duplicated region was lost in 11 of the tetrads. In these tetrads all 4 ascospores had a normalized sequencing coverage of the duplicated region of approximately 1 (Table S3B), while in 12 tetrads the duplication was still present (normalized sequencing coverage of approximately 2) and therefore followed Mendelian segregation. In 10 of these 12 tetrads that still had the duplication, RIP mutations were detectable in this region, while in the remaining 2 tetrads no RIP mutations occurred in this region (tetrads A03-9 and S1C4_A2) (Fig. S5A). The duplicated region contains six predicted genes, one of which has a functional annotation describing it as similar to transferase hexapeptide domain protein. Therefore, the putative function of the genes affected by the RIP of the duplication cannot yet be deduced.

10.1128/mbio.03290-22.4 TABLE S3 **A** List of all meiotic *de novo* mutations **B** Overview of tetrads, spores, mapping results, and the number of high-fidelity SNPs. Download Table S3, XLSX file, 0.1 MB. Copyright © 2023 Komluski et al. 2023 Komluski et al. https://creativecommons.org/licenses/by/4.0/ This content is distributed under the terms of the Creative Commons Attribution 4.0 International license.

10.1128/mbio.03290-22.9 FIG S5 Distribution and segregation of *de novo* mutations. (A) Number of *de novo* mutations per tetrad. Blue shaded bars display the number of CG:TA transitions on 14 kb duplication on chromosome 3, inside TEs and outside TEs per tetrad. Red bars depict the number of TA:CG transitions and transversions. “D” indicates tetrads in which the 14 kb duplication is present in two of the four ascospores of the ascus. (B) Distribution of meiotic SNPs along different classes of TEs. Each TE was divided into 40 equal windows. Each black rectangle on the *x* axis represents a window inside a TE representing 2.5% of the TE length. Beige rectangles represent windows in the regions directly adjacent to TEs. Dots above rectangles represent one mutation in each window (yellow dots, AT:GC transitions; blue dots, CG:TA transitions, red dots, transversions). (C) Segregation of *de novo* mutations in different genomic compartments. Light red bars show the relative frequency of *de novo* mutations with 1:3 segregation, and grey bars display the relative frequency of *de novo* mutations with 2:2 segregation for the different genomic compartments. Fisher’s exact test *P* values are shown (*, *P < *0.05; **, *P < *0.005; ***, *P < *0.0005). (D) Dinucleotide context of the 242 mutations within the 14 kb duplication on chromosome 3. The relative fraction (in ‰) of the dinucleotide sites that were ripped are depicted. (E) Variation of the mutation rate and the relative fraction CpA dinucleotides along the 14 kb duplication on chromosome 3 in sliding 500 bp windows. (F) Distribution of the number of RIP mutations within those ascospores that contained the duplication on chromosome 3 (marked “D” in panel A). Download FIG S5, TIF file, 2.2 MB. Copyright © 2023 Komluski et al. 2023 Komluski et al. https://creativecommons.org/licenses/by/4.0/ This content is distributed under the terms of the Creative Commons Attribution 4.0 International license.

We further addressed to what extent *de novo* mutations could be the result of meiosis-associated RIP. Here, we found that 172 of the meiotic *de novo* mutations (32% of all *de novo* mutations) were detected in transposable elements (Fig. 3C), and 166 of these 172 mutations (96.5%) were CG:TA transitions, the putative signature of RIP. To examine whether these mutations were also most likely caused by RIP, we determined which class of transposable elements was affected. Of the 172 *de novo* mutations in TEs, 152 (88.3%) were located in class I transposons, which replicate via an RNA intermediate and are also referred to as copy-and-paste transposons (70–72). Of these 152 mutations in class I transposons (long interspersed nuclear elements [LINEs] and long terminal repeats [LTRs]), 146 were CG:TA transitions, with 5 TA:GC transitions and 1 G→C transversion, while only 11 CG:TA transitions occurred in class II transposable elements (Fig. 3D). Class II TEs are referred to as cut-and-paste transposons, which are excised and moved to new locations in the genome and hence do not create repeated sequences (71). The mutation rate in class I (copy-and-paste) TEs is 1.3 × 10^−6^ per bp per generation compared to 2.8 × 10^−7^ per bp per generation for class II (cut-and-paste) TEs, with the vast majority of the *de novo* mutations showing RIP-like CG:TA transitions.

Investigations in a few other ascomycete fungi have provided evidence that RIP can affect the vicinity of duplicated regions by “leakage” of mutations into these non-repeated regions (53, 54). Based on these previous studies, we here also asked if RIP mutations would be present in regions adjacent to TEs. We subdivided each TE into 40 equally sized windows (average window size, 71 bp) and the two adjacent regions into 20 windows each (window size, 71 bp) and counted the number of *de novo* mutations in each of these windows. This approach allowed us to show that RIP mutations were not equally distributed along TE sequences. Within TEs the most distal windows showed the highest number of *de novo* mutations, whereas the more central sequences were less likely to be mutated (Fig. 3E). Further analysis of the effect of the different classes of transposable elements showed that the highest number of RIP mutations in the distal regions of the TEs was mainly due to LTR transposons (Fig. S5B), whereas LINE and class II transposable elements did not show such a variation of the number of mutations along the length of the transposable element (Fig. S5B). In regions adjacent to the TEs a much lower number of mutations occurred than occurred in the TE sequences. Overall, the mutation rate in the regions adjacent to TEs was 7.4 × 10^−8^ per bp per generation, which was lower than the genome-wide mutation rate of 1.5 × 10^−7^ per bp per generation (for regions excluding TEs and excluding the duplication). It therefore appears that leakage has not occurred in the vicinity of the RIP-mutated TEs. Only 21.3% (112 mutations) of all *de novo* mutations were located in the regions outside the transposable elements and the 14 kb region on chromosome 3. These *de novo* mutations in non-TE and non-duplicated regions amounted to a significantly higher proportion of TA:CG transitions and transversions (22.3%) (Fig. 3C).

The segregation pattern of the *de novo* mutations varied between the different compartments, with those in TEs showing a higher proportion of 1:3 segregation (Fig. S5C) than those in the 14 kb duplication or the mutations outside TEs. The segregation pattern of *de novo* mutations can indicate at what stage a DNA lesion or mismatch may have occurred and at what stage this lesion or mismatch (possibly caused by RIP) was resolved. A 2:2 segregation indicates that the mismatch was resolved prior to the replication cycle of the meiosis, while a 1:3 segregation indicates that the mismatch was resolved only during replication or later. Hence, the higher proportion of 1:3 segregation patterns observed in the TEs could indicate a delayed resolution of the mismatches introduced by RIP in the heterochromatic TEs compared to the resolution in other genomic compartments (Fig. S1A). The 3′ neighbor of the targeted cytosine has a strong influence on RIP in N. crassa, with a strong preference for CpA (73, 74). We therefore identified the dinucleotide context of the 242 mutations in the 14 kb duplicated regions. Of these, 240 were in a CpA context, and the remaining two were in CpT context. The rate at which a single CpA was mutated during RIP was therefore 0.54% (Fig. S5D). The mutation rate varied along the 14 kb duplication, but this did not appear to correlate with the proportion of dinucleotides in the sequence that were CpA (Fig. S5E). The number of individual mutations that were induced within the duplicated region varied considerably between tetrads (Fig. S5A) but also between the individual ascospores of the tetrads (Fig. S5F). For the 24 ascospores containing the duplication on chromosome 3, the number of mutations within the duplication in individual ascospores varied from 0 to a maximum of 57 (median 11), with most ascospores showing 0 to 10 mutations (Fig. S5F). In summary, RIP has been proposed to be an important player in the genome evolution of *Z. tritici*. However, this is the first experimental evidence for active RIP in this fungus, and we observed that the large duplication was not always mutated by the RIP mechanism and that the total number of mutations introduced by RIP was low—leaving many cytosines in the duplicated regions unaffected—which together could indicate a low efficiency of RIP in *Z. tritici*.

## DISCUSSION

Here, we used tetrad analysis to estimate recombination rates, gene conversion rates, and *de novo* mutation rates associated with meiosis from 23 individual meioses in *Z. tritici*. The ability to dissect genetic events in individual ascospore progenies isolated from tetrads provided us with highly precise maps of meiosis-associated changes along the fungal genome. Our results show (i) higher gene conversion rates and recombination rates on accessory chromosomes compared to core chromosomes, (ii) a correlation of recombination rates and gene conversion rates with histone marks associated with heterochromatin, and (iii) elevated *de novo* mutation rates during sexual reproduction caused by active RIP.

Our dissection of recombination events during individual meiotic events allows us to confirm the previously reported high recombination rates in *Z. tritici*—on average, 65 cM/Mb. Interestingly, 35 of the 52 recombination hotspots detected in this study overlapped with previously reported recombination hotspots in *Z. tritici*, which used distinct isolates from Switzerland, indicating that recombination hotspots may be determined by certain domains or conserved marks along the genome (17). However, these are most likely not small sequence motifs, as there is little similarity between those identified within our study and those from the previous study with Swiss isolates (17). In contrast to previous studies using population genomic data (18), we show that the rate of recombination is, in fact, higher on accessory chromosomes than on core chromosomes (92.7 cM/Mb and 62.6 cM/Mb, respectively) and is negatively correlated with chromosome size. Hence, our results are in line with the general observation that smaller chromosomes tend to have higher recombination rates (75). Earlier population-based recombination rate studies in *Z. tritici* showed, however, lower recombination rates computed as *rho* for accessory chromosomes (*rho *= 0.001) than on core chromosomes (*rho *= 0.024) (18). *Rho* is the product of the actual recombination rate and the effective population size Ne, and we speculate that the discrepancy in recombination rate measures may reflect the lower effective population size of the accessory chromosomes compared to the core chromosomes (18). Intriguingly, we could not identify any crossovers on an accessory chromosome in nine instances, despite the presence of the respective homologous chromosomes. A minimum of one crossover per homologous chromosome pair is considered to be required for proper segregation of the homologous chromosomes (4, 76). Indeed, in four of these nine instances, we observed such segregation errors. However, in the remaining five instances, the accessory chromosomes properly segregated despite the absence of crossovers. Hence, we conclude that crossovers are not essential for proper chromosome segregation in *Z. tritici*. *spo11* deletion mutants in S. cerevisiae and Sordaria macrospora were previously used to observe the consequences of the absence of DSBs and recombination on the segregation of chromosomes during meiosis. In these fungal species the absence of DSBs and recombination caused widespread chromosome segregation errors, highlighting the importance of recombination for proper segregation (77, 78). The relatively high frequency of properly segregated, non-recombined chromosomes in *Z. tritici* indicates the presence of a non-recombination-dependent segregation system which might also be involved in the meiotic drive system of the accessory chromosomes in *Z. tritici* (60).

We predict that high levels of gene conversions in *Z. tritici* can affect allele frequencies to a greater extent than recombination and thereby shape genome composition. The genome-wide gene conversion rate of 1.6 × 10^−3^ per SNP identified in our study is approximately 20 times lower than the genome-wide gene conversion rate in S. cerevisiae (3.8 × 10^−2^ per SNP) (32, 33), but approximately an order of magnitude higher than the genome-wide gene conversion rate in N. crassa (0.7 × 10^−4^ to 2.2 × 10^−4^ per SNP) (32, 33). The variation in the gene conversion rate between species might be influenced by tract length and recombination rate since gene conversion is positively correlated with both characteristics (33, 34, 36). Our data confirm this since *Z. tritici* has shorter NCO-GC and CO-GC tract lengths and lower recombination rates (539 bp, 432 bp, and 65 cM/Mb, respectively) than S. cerevisiae (1,681 bp, 1,841 bp, and 375 cM/Mb) (33). N. crassa in turn shows longer NCO-GC tract lengths but shorter CO-GC tract lengths and a lower recombination rate (950 bp, 284 bp, and 20 cM/Mb, respectively) than *Z. tritici* (33). Similar to recombination, accessory chromosomes have higher gene conversion rates than the core chromosomes. The smaller size of accessory chromosomes in contrast to core chromosomes could influence the gene conversion rate on accessory chromosomes since smaller chromosomes tend to have higher rates of gene conversion (33).

We found a strong association between chromatin modifications and recombination and gene conversion rates. We speculate that this might be due to two non-exclusive mechanisms: the effect of histone modifications on the location of DSBs and/or their effect on DSB and heteroduplex repair. Although a direct correlation between DSB formation and H3K4me3 has been shown in S. cerevisiae (20), the underlying causality of this correlation and the extent to which it applies to other organisms is unclear (79, 80). Local chromatin conformation can influence how DSBs and heteroduplex DNA are repaired, thereby influencing the later stages leading to recombination and gene conversion (81–83). In contrast to S. cerevisiae, we see an association of heterochromatin as marked by H3K9me3 and H3K27me3 with recombination in *Z. tritici*. Similarly, and again in contrast to the scenario in yeast, we observe that gene conversion rates are highest in heterochromatin regions in *Z. tritici*. This suggests that chromatin conformation may affect gene conversion primarily through its effect on DSB and heteroduplex repair and not via its impact on accessibility. DSBs can be resolved by synthesis-dependent strand annealing (SDSA), leading to a homologous recombination-mediated pathway and hence NCO events (84, 85). Our results show increased gene conversion rates in regions enriched in H3K9me3. Several studies suggest that H3K9 di- or trimethylated (H3K9me2/3) heterochromatin promotes homologous recombination (HR) (85–89). DNA double-strand breaks promote chromatin stabilization by H3K9me3 and activate DSB signaling proteins (90). The role of H3K9me3 in promoting homologous recombination could mean that the correlation between H3K9me3 and the gene conversion rate we observed in our study is caused by the effect of H3K9me3 on recombination. However, 80% of the gene conversion events we detected are associated with non-crossover events (NCO-GC). This suggests that the correlation between H3K9me3 and gene conversions is caused not by recombination, but possibly by the effect of histone modifications on the repair of DSBs and heteroduplex DNA. In contrast to DSBs in regions enriched in H3K9me3, DSBs in regions enriched in H3K27me3 were found to be frequently repaired by microhomology-mediated end joining (MMEJ), a nonhomologous repair pathway that does not promote homologous recombination and therefore does not lead to gene conversion or crossover (91, 92). We find support for a similar effect of chromatin modifications on DSB repair in our study. Indeed, increased repair of DSBs via a nonhomologous pathway in the H3K27me3-enriched regions could explain the reduced gene conversion rate in the regions enriched in both the H3K9me3 and H3K27me3 regions (1.3 × 10^−3^ per SNP) compared to regions enriched in H3K9me3 only (2.8 × 10^−3^ per SNP) as observed here. Similarly, the absence of an increased gene conversion rate despite the high frequency of crossovers in the regions enriched in H3K27me3 alone could be explained by MMEJ being favored by H3K27me3. However, it is unclear why H3K27me3 is associated with the highest crossover frequencies, since MMEJ should not promote homologous recombination and therefore should result in a lower crossover frequency. In wheat, high crossover rates were associated with H3K27me3, but the mechanism causing this correlation is still unclear (93). We speculate that the observed correlation between H3K27me3 and higher crossover frequencies may be driven by the enrichment of this mark on accessory chromosomes, but functional validation is still missing. In conclusion, we see indications that histone modifications could affect gene conversion rates mainly via their effect on the DSB and heteroduplex repair.

*De novo* mutations associated with meiosis occurred at a rate of 5.7 × 10^−7^ per bp per generation in *Z. tritici*, which is approximately three orders of magnitude higher than the mitotic mutation rate (3.2 × 10^−10^ per site per cell division) which we previously determined in a mutation accumulation experiment (59). *De novo* mutations were not uniformly distributed across the *Z. tritici* genome, ranging from the highest mutation rate of 7.5 × 10^−4^ per bp per generation within the duplicated region on chromosome 3 to 1.5 × 10^−7^ per bp per generation in genomic regions excluding the duplicated region and excluding TEs. Class I and II TEs showed a mutation rate of 1.3 × 10^−6^ and 2.8 × 10^−7^ per bp per generation, respectively, which is higher than the genome mutation rate but lower than the mutation rate in the duplicated region. This is probably due to the variability of TEs within these two groups, which also contain many non-duplicated sequences. Higher meiotic than mitotic mutation rates have been reported in other fungi such as S. cerevisiae and N. crassa (44, 46–48). In N. crassa, the difference between mutation rates during mitosis and meiosis is mostly due to RIP, a fungal defense mechanism againt duplicated DNA sequences that occurs in the haploid nuclei just prior to meiosis and that induces CG:TA transitions in duplicated sequences and transposable elements (47, 50). In *Z. tritici*, signatures of past RIP have been found by analyses of genome data (56, 94), and here we can confirm that RIP is an active mechanism in *Z. tritici*. This is evident as 77% of the *de novo* mutations associated with meiosis were located in class I transposable elements (copy-and-paste elements) and in the 14,001 bp long region on chromosome 3 that was duplicated in the IPO94269 parent. Interestingly, RIP in *Z. tritici* is not consistently efficient in mutating duplicated sequences, as we find that duplicated sequences in two of the tetrads were not mutated and the number of RIP mutations introduced even in duplicated regions is low. The duplication that was present in the parental strain IPO94269 appears to have been frequently lost during crosses. How and at what stage this loss of the duplication occurred is currently unclear. However, most likely, the loss must have occurred before the RIP process, as no RIP mutations were detected in this region. Until now, RIP was experimentally demonstrated in only a few fungal species (95–100), with lower efficiency in Leptosphaeria maculans and Podospora anserina than in the highly efficient RIP in N. crassa (97, 101). We consider that variation in RIP efficiency may be a more common phenomenon that reflects a trade-off between the evolutionary costs of the mutations introduced by RIP and the evolutionary costs of TEs.

We find that class I (copy-and-paste) TEs show a much higher RIP mutation rate than class II (cut-and-paste). This observation is expected, as the transposition mode of the class I TEs results in duplicated sequences that are recognized by the RIP machinery (70). Interestingly, we find that the RIP mutations are not equally distributed along the TE sequences. The most distal windows composing 5% of the length of the TEs amount to 35% of all *de novo* meiotic mutations that occurred within the TEs. This high frequency of *de novo* mutations at the ends of TEs was mostly due to the accumulation of mutations in the distal parts of the LTR transposons. LTR transposons contain terminal repeats of 200 bp to 500 bp in length and therefore already contain duplicated sequences in close vicinity (72). These duplicated terminal sequences in class I transposons seem to be prominent targets for RIP. In various other fungi, RIP leaks into adjacent regions from the duplicated sequences of TEs (53, 102, 103) and is thought to play a role in the rapid adaptive evolution of effector genes involved in host-pathogen interactions (53, 102, 103). However, we do not see such leakage from transposable elements in *Z. tritici* but, rather, see lower mutation rates in the vicinity of transposable elements than in the rest of the genome, which we cannot currently explain. Taking these data together, we show that RIP affects transposable elements in *Z. tritici* to various degrees and that the mutational environment thus influences the activity of these important drivers of genome evolution.

In conclusion, we show that recombination and gene conversion are correlated with histone modifications in different ways in *Z. tritici* and that RIP is active, albeit at a lower efficiency than in N. crassa, in this fungus, affecting duplicated sequences as well as TEs. As a result, meiotic mutation rates for *Z. tritici* are three orders of magnitude higher than the mitotic rates, demonstrating the major impact that genetic changes associated with meiosis have on the genome composition of this important plant pathogen.

## MATERIALS AND METHODS

### Fungal material.

Tetrads used for the sequencing analysis were obtained from the study of Habig et al. (60) and include ascospores isolated from crosses between the Dutch isolates IPO94269 and IPO323 (available from the Westerdijk Institute, Utrecht, The Netherlands, with the accession numbers CBS115943 and CBS115941) and from the crosses between IPO94269 and whole-chromosome isogenic deletion strains (Δchr14 Δchr20) of the reference strain IPO323 generated in the study of Habig et al. (57). All ascospores were cultivated for DNA extraction at 18°C and 200 rpm in YMS (4 g/L yeast extract, 4 g/L malt, 4 g/L sucrose) medium for 5 to 7 days inoculated directly from −80°C glycerol stocks.

### Genome sequencing and data analysis.

For sequencing, DNA of 84 ascospores was isolated using a phenol-chloroform extraction protocol as described previously (60). Eight ascospores were available per tetrad due to an additional mitosis that follows meiosis II in *Z. tritici*. A previous study, which sequenced all eight ascospores of two tetrads, confirmed that each tetrad contained four twins as the result of the mitosis (60). Based on the molecular characterization of all ascospores of the 23 tetrads from the previous study (60), we selected four unique ascospores for each tetrad for sequencing. Library preparation and sequencing using an Illumina HiSeq 3000 machine for the 84 ascospores was performed at the Max Planck-Genome-Center, Cologne, Germany. The Illumina read data are available in the Sequence Read Archive under BioProject number PRJNA904559. Please note that two tetrads (A03-4 and A08-1) that were sequenced in an earlier study were also included (60). The Illumina read data for these two tetrads are available at the Sequence Read Archive under BioProject number PRJNA438050. An overview of the included tetrads and ascospores is given in Table S3B. We visually checked the distribution of parental haplotypes within each tetrad. Only 23 tetrads showing a 2:2 segregation of parental haplotypes and crossover events involving the homologous chromosome of two ascospores were included (see example in Fig. S6). One of the original 24 tetrads was excluded from the analysis because it showed a pattern of parental haplotypes on multiple chromosomes that was inconsistent with meiotic recombination and was thought to be not the product of a single meiosis but, rather, a mixture of two or more meiotic events. In addition, the parental strain IPO94269 was sequenced as described above, and its Illumina reads were deposited under the BioProject number PRJNA904744. The Illumina data for the IPO323 parental strains are available under BioProject number PRJNA371572.

10.1128/mbio.03290-22.10 FIG S6 Example of the distribution of parental haplotypes within the four ascospores of the A03-9 tetrad. Blue and red represent the haplotype of the parental strains IPO323 and IPO94269, respectively. Note that the parental haplotypes show a 2:2 segregation, and crossover events involve the homologous chromosome of two ascospores. Note that chromosome 18 (chr18) and chr20 are not present in IPO94269 and were therefore removed from the analysis. Download FIG S6, TIF file, 2.3 MB. Copyright © 2023 Komluski et al. 2023 Komluski et al. https://creativecommons.org/licenses/by/4.0/ This content is distributed under the terms of the Creative Commons Attribution 4.0 International license.

### Mapping and SNP calling.

The reads of 92 ascospores of the 23 tetrads were mapped to the IPO323 reference genome with Bowtie 2 (version 2.3.4.1) (104). This analysis focused only on single nucleotide polymorphisms (SNPs). To obtain a high-quality SNP data set, we performed the SNP calling with two variant callers, GATK (version 4.1.6.0) (105) and SAMtools (version 1.7) (106), and SNPs with a QUAL value of ≥90 that were called by both variant callers were used for the downstream processing. Variants in regions that contain transposable elements (TEs) were removed from the analysis with BEDTools Intersect (version 2.26.0, option -v) (107) to avoid spurious alignments. From the remaining SNPs, only variants from regions with coverage of >5 in all four ascospores of a tetrad were used for the analysis to avoid false negatives due to the low coverage in one of the spores. Biallelic SNPs with minor allele frequency of >0.9 and with QUAL of >90 that were called by both variant callers were identified by overlapping variant call format (VCF) files from both haplotype callers with BEDTools Intersect and used as a core set of high-fidelity SNPs. VCF files of four spores from the same tetrad were merged with VCFtools (v0.1.15) with the merge option (108) to create a variant file for each tetrad.

High-quality threshold and variant calling accepting only variants identified by two callers will potentially lead to false-negative calls in some of the four spores and hence will affect the segregation ratio obtained for the SNPs. To reduce the risk of false-negative calls, we reintroduced high-fidelity SNPs to any of the other ascospores of a tetrad if there was an indication that it was present but did not satisfy the quality requirements. This means that SNPs which were called in a tetrad but did not meet the quality threshold in some of the ascospores but met the quality threshold in at least one of the ascospores were reintroduced. SNPs on chromosomes that were deleted or absent in the parental strains were removed from the analysis of the respective progeny. Please see Table S3B for an overview of the number of high-fidelity SNPs included for each tetrad.

### Identification of recombination events.

To detect recombination and gene conversion events in the tetrad progeny, CrossOver.py from the ReCombine package (version 2.1) for tetrad analysis in yeast (64) was modified to fit the genome characteristics of *Z. tritici* (size and number of chromosomes and the location of the centromeres [58]). Input segregation files for the CrossOver program were generated from merged tetrad VCF files for each tetrad with the custom-made bash script (see Text S1). Each segregation file consisted of seven columns: the first two columns referred to the chromosome and position of the variant, the third column served as a spacer, and the last four columns referred to the presence/absence of SNPs in four spores. Values of 0 and 1 in the last four columns of a segregation file designated the presence or absence of a variant at a certain position compared to the reference genome. The program initially identifies COs as positions with 2:2 segregation where adjacent markers undergo a reciprocal genotype switch. Gene conversion tracts are then identified as regions of non-2:2 segregation. After the identification of recombination events, all double crossovers separated with a single SNP were filtered out. Gene conversions were filtered for tracts spanning ≥3 markers. The recombination rate per tetrad (cM/Mb) was calculated with the following formula:

recombination rate = (½ × number of CO × 100)/genome size

10.1128/mbio.03290-22.1 TEXT S1 Description of the bioinformatic procedures and tools and Sanger sequencing results. Download Text S1, PDF file, 0.1 MB. Copyright © 2023 Komluski et al. 2023 Komluski et al. https://creativecommons.org/licenses/by/4.0/ This content is distributed under the terms of the Creative Commons Attribution 4.0 International license.

The gene conversion rate per tetrad was determined as the proportion of converted markers from the total number of markers identified per tetrad. Furthermore, tract lengths were determined with the midpoint method, i.e., the midpoint between two markers of a different class (e.g., converted versus non-converted) was considered to be the position were the tract started or ended. Tracts spanning TEs were removed for the estimation of tract lengths and recombination rates. Recombination events and gene conversion events detected in this study are listed in the supplemental material (Table S1B and C and Table S2A to C, respectively). SNPs in TEs were disregarded for the determination of recombination and gene conversion events.

### Estimation of meiotic mutation rates.

To estimate meiotic mutation rates in *Z. tritici*, genome-wide SNPs (including SNPs in TEs) satisfying the following criteria were taken into consideration: (i) read depth of >5 in both parental strains and the ascospore progeny and (ii) absent in both parental strains (Table S3A). Before a SNP in the progeny was considered a *de novo* mutation, both parental sequencing results were manually checked to validate that this SNP was already present but not called in the parental genomes. Only SNPs in the progeny that showed no hints in the parental genomes were included in the subsequent analysis. The per bp mutation rate was calculated as the average number of meiotic mutations per ascus/the reference genome size. To verify *in silico*-detected meiotic mutations, we performed Sanger sequencing of 20 randomly selected mutations, from which 19 mutations were confirmed (see Text S1).

### Detection of duplications.

For the detection of duplications in the parental strains, Illumina reads were quality filtered as described above and mapped onto the reference genome using SpeedSeq align followed by structural variation analysis using LUMPY (109) as implemented in the SpeedSeq package (version 0.1.2) (110). The VCF files were filtered using BCFtools (version 1.6) as follows: VCF files were filtered on duplications, genotype (GT, 0/1), quality of >400, and length of <50,000.

### Correlation analysis with genomic features.

The following genomic compartments were considered, as these have been previously determined: histone modification enrichment (H3K4me2, H3K9me3, H3K27me3) as defined in reference 58, gene models (63), and transposable elements (111). Simple sequence repeats (SSR) were annotated *ab initio* using GMATA (version 2.3) (112).

For motif discovery in the vicinity of crossover events, 500 bp upstream and downstream genomic sequence surrounding the individual crossover events were extracted from the genome and used for motif discovery with HOMER (version 4.11) (113). The four most significant *de novo*-identified motifs with a *P* value of < 1 × 10^−15^ were selected.

### Data availability.

The Illumina read data are available in the Sequence Read Archive under the BioProjects PRJNA904559, PRJNA438050, PRJNA904744, and PRJNA371572. The *Z. tritici* IPO323 reference genome is available under accession number GCA_000219625.1.

## References

[1] Peñalba JV, Wolf JBW. 2020. From molecules to populations: appreciating and estimating recombination rate variation. Nat Rev Genet 21:476–492. doi:10.1038/s41576-020-0240-1. 32472059

[2] Keeney S. 2008. Spo11 and the formation of DNA double-strand breaks in meiosis, p 81–123. *In* Recombination and meiosis. Springer Berlin Heidelberg, Berlin, Heidelberg. 10.1007/7050_2007_026 PMC3172816 21927624

[3] Zelkowski M, Olson MA, Wang M, Pawlowski W. 2019. Diversity and determinants of meiotic recombination landscapes. Trends Genet 35:359–370. doi:10.1016/j.tig.2019.02.002. 30948240

[4] Zickler D, Kleckner N. 2015. Recombination, pairing, and synapsis of homologs during meiosis. Cold Spring Harb Perspect Biol 7:a016626. doi:10.1101/cshperspect.a016626. 25986558 PMC4448610

[5] Chen J-M, Cooper DN, Chuzhanova N, Férec C, Patrinos GP. 2007. Gene conversion: mechanisms, evolution and human disease. Nat Rev Genet 8:762–775. doi:10.1038/nrg2193. 17846636

[6] Kleckner N. 1996. Meiosis: how could it work? Proc Natl Acad Sci USA 93:8167–8174. doi:10.1073/pnas.93.16.8167. 8710842 PMC38641

[7] Korunes KL, Noor MAF. 2017. Gene conversion and linkage: effects on genome evolution and speciation. Mol Ecol 26:351–364. doi:10.1111/mec.13736. 27337640

[8] Youds JL, Boulton SJ. 2011. The choice in meiosis: defining the factors that influence crossover or non-crossover formation. J Cell Sci 124:501–513. doi:10.1242/jcs.074427. 21282472

[9] Henderson IR, Bomblies K. 2021. Evolution and plasticity of genome-wide meiotic recombination rates. Annu Rev Genet 55:23–43. doi:10.1146/annurev-genet-021721-033821. 34310193

[10] Bergero R, Ellis P, Haerty W, Larcombe L, Macaulay I, Mehta T, Mogensen M, Murray D, Nash W, Neale MJ, O’Connor R, Ottolini C, Peel N, Ramsey L, Skinner B, Suh A, Summers M, Sun Y, Tidy A, Rahbari R, Rathje C, Immler S. 2021. Meiosis and beyond: understanding the mechanistic and evolutionary processes shaping the germline genome. Biol Rev Camb Philos Soc 96:822–841. doi:10.1111/brv.12680. 33615674 PMC8246768

[11] Munz P. 1994. An analysis of interference in the fission yeast Schizosaccharomyces pombe. Genetics 137:701–707. doi:10.1093/genetics/137.3.701. 8088515 PMC1206029

[12] Mercier R, Mézard C, Jenczewski E, Macaisne N, Grelon M. 2015. The molecular biology of meiosis in plants. Annu Rev Plant Biol 66:297–327. doi:10.1146/annurev-arplant-050213-035923. 25494464

[13] Blary A, Jenczewski E. 2019. Manipulation of crossover frequency and distribution for plant breeding. Theor Appl Genet 132:575–592. doi:10.1007/s00122-018-3240-1. 30483818 PMC6439139

[14] Haenel Q, Laurentino TG, Roesti M, Berner D. 2018. Meta-analysis of chromosome-scale crossover rate variation in eukaryotes and its significance to evolutionary genomics. Mol Ecol 27:2477–2497. doi:10.1111/mec.14699. 29676042

[15] Choi K, Henderson IR. 2015. Meiotic recombination hotspots: a comparative view. Plant J 83:52–61. doi:10.1111/tpj.12870. 25925869

[16] Lichten M, Goldman ASH. 1995. Meiotic recombination hotspots. Annu Rev Genet 29:423–444. doi:10.1146/annurev.ge.29.120195.002231. 8825482

[17] Croll D, Lendenmann MH, Stewart E, McDonald BA. 2015. The impact of recombination hotspots on genome evolution of a fungal plant pathogen. Genetics 201:1213–1228. doi:10.1534/genetics.115.180968. 26392286 PMC4649646

[18] Stukenbrock EH, Dutheil JY. 2018. Fine-scale recombination maps of fungal plant pathogens reveal dynamic recombination landscapes and intragenic hotspots. Genetics 208:1209–1229. doi:10.1534/genetics.117.300502. 29263029 PMC5844332

[19] Sardell JM, Cheng C, Dagilis AJ, Ishikawa A, Kitano J, Peichel CL, Kirkpatrick M. 2018. Sex differences in recombination in Sticklebacks. G3 (Bethesda) 8:1971–1983. doi:10.1534/g3.118.200166. 29632132 PMC5982825

[20] Pan J, Sasaki M, Kniewel R, Murakami H, Blitzblau HG, Tischfield SE, Zhu X, Neale MJ, Jasin M, Socci ND, Hochwagen A, Keeney S. 2011. A hierarchical combination of factors shapes the genome-wide topography of yeast meiotic recombination initiation. Cell 144:719–731. doi:10.1016/j.cell.2011.02.009. 21376234 PMC3063416

[21] Borde V, Robine N, Lin W, Bonfils S, Géli V, Nicolas A. 2009. Histone H3 lysine 4 trimethylation marks meiotic recombination initiation sites. EMBO J 28:99–111. doi:10.1038/emboj.2008.257. 19078966 PMC2634730

[22] Buard J, Barthès P, Grey C, de Massy B. 2009. Distinct histone modifications define initiation and repair of meiotic recombination in the mouse. EMBO J 28:2616–2624. doi:10.1038/emboj.2009.207. 19644444 PMC2738703

[23] Choi K, Zhao X, Kelly KA, Venn O, Higgins JD, Yelina NE, Hardcastle TJ, Ziolkowski PA, Copenhaver GP, Franklin CH, Mcvean G, Henderson IR. 2013. Arabidopsis meiotic crossover hot spots overlap with H2A.Z nucleosomes at gene promoters. Nat Genet 45:1327–1336. doi:10.1038/ng.2766. 24056716 PMC3812125

[24] Underwood CJ, Choi K, Lambing C, Zhao X, Serra H, Borges F, Simorowski J, Ernst E, Jacob Y, Henderson IR, Martienssen RA. 2018. Epigenetic activation of meiotic recombination near Arabidopsis thaliana centromeres via loss of H3K9me2 and non-CG DNA methylation. Genome Res 28:519–531. doi:10.1101/gr.227116.117. 29530927 PMC5880242

[25] Fouché S, Plissonneau C, McDonald BA, Croll D. 2018. Meiosis leads to pervasive copy-number variation and distorted inheritance of accessory chromosomes of the wheat pathogen Zymoseptoria tritici. Genome Biol Evol 10:1416–1429. doi:10.1093/gbe/evy100. 29850789 PMC6007412

[26] Chan AH, Jenkins PA, Song YS. 2012. Genome-wide fine-scale recombination rate variation in Drosophila melanogaster. PLoS Genet 8:e1003090. doi:10.1371/journal.pgen.1003090. 23284288 PMC3527307

[27] Daugherty MD, Zanders SE. 2019. Gene conversion generates evolutionary novelty that fuels genetic conflicts. Curr Opin Genet Dev 58–59:49–54. doi:10.1016/j.gde.2019.07.011. PMC6889005 31466040

[28] Lazzaro BP, Clark AG. 2001. Evidence for recurrent paralogous gene conversion and exceptional allelic divergence in the Attacin genes of Drosophila melanogaster. Genetics 159:659–671. doi:10.1093/genetics/159.2.659. 11606542 PMC1461832

[29] Thomas JH. 2006. Concerted evolution of two novel protein families in Caenorhabditis species. Genetics 172:2269–2281. doi:10.1534/genetics.105.052746. 16415360 PMC1456376

[30] Buchmann K. 2014. Evolution of innate immunity: clues from invertebrates via fish to mammals. Front Immunol 5:459. doi:10.3389/fimmu.2014.00459. 25295041 PMC4172062

[31] Lorenz A, Mpaulo SJ. 2022. Gene conversion: a non-Mendelian process integral to meiotic recombination. Heredity 129:56–63. doi:10.1038/s41437-022-00523-3. 35393552 PMC9273591

[32] Mancera E, Bourgon R, Brozzi A, Huber W, Steinmetz LM. 2008. High-resolution mapping of meiotic crossovers and non-crossovers in yeast. Nature 454:479–485. doi:10.1038/nature07135. 18615017 PMC2780006

[33] Liu H, Huang J, Sun X, Li J, Hu Y, Yu L, Liti G, Tian D, Hurst LD, Yang S. 2017. Tetrad analysis in plants and fungi finds large differences in gene conversion rates but no GC bias. Nat Ecol Evol 2:164–173. doi:10.1038/s41559-017-0372-7. 29158556 PMC5733138

[34] Mansai SP, Kado T, Innan H. 2011. The rate and tract length of gene conversion between duplicated genes. Genes 2:313–331. doi:10.3390/genes2020313. 24710193 PMC3924818

[35] Lesecque Y, Mouchiroud D, Duret L. 2013. GC-biased gene conversion in yeast is specifically associated with crossovers: molecular mechanisms and evolutionary significance. Mol Biol Evol 30:1409–1419. doi:10.1093/molbev/mst056. 23505044 PMC3649680

[36] Marais G. 2003. Biased gene conversion: implications for genome and sex evolution. Trends Genet 19:330–338. doi:10.1016/S0168-9525(03)00116-1. 12801726

[37] Pessia E, Popa A, Mousset S, Rezvoy C, Duret L, Marais GAB. 2012. Evidence for widespread GC-biased gene conversion in eukaryotes. Genome Biol Evol 4:675–682. doi:10.1093/gbe/evs052. 22628461 PMC5635611

[38] Arbeithuber B, Betancourt AJ, Ebner T, Tiemann-Boege I. 2015. Crossovers are associated with mutation and biased gene conversion at recombination hotspots. Proc Natl Acad Sci USA 112:2109–2114. doi:10.1073/pnas.1416622112. 25646453 PMC4343121

[39] Arbel-Eden A, Simchen G. 2019. Elevated mutagenicity in meiosis and its mechanism. BioEssays 41:1800235. doi:10.1002/bies.201800235. 30920000

[40] Galagan JE, Selker EU. 2004. RIP: the evolutionary cost of genome defense. Trends Genet 20:417–423. doi:10.1016/j.tig.2004.07.007. 15313550

[41] Lynch M, Ackerman MS, Gout J-F, Long H, Sung W, Thomas WK, Foster PL. 2016. Genetic drift, selection and the evolution of the mutation rate. Nat Rev Genet 17:704–714. doi:10.1038/nrg.2016.104. 27739533

[42] Narasimhan VM, Rahbari R, Scally A, Wuster A, Mason D, Xue Y, Wright J, Trembath RC, Maher ER, van Heel DA, Auton A, Hurles ME, Tyler-Smith C, Durbin R. 2017. Estimating the human mutation rate from autozygous segments reveals population differences in human mutational processes. Nat Commun 8:303. doi:10.1038/s41467-017-00323-y. 28827725 PMC5566399

[43] Rahbari R, Wuster A, Lindsay SJ, Hardwick RJ, Alexandrov LB, Al Turki S, Dominiczak A, Morris A, Porteous D, Smith B, Stratton MR, Hurles ME, UK10K Consortium. 2016. Timing, rates and spectra of human germline mutation. Nat Genet 48:126–133. doi:10.1038/ng.3469. 26656846 PMC4731925

[44] Rattray A, Santoyo G, Shafer B, Strathern JN. 2015. Elevated mutation rate during meiosis in Saccharomyces cerevisiae. PLoS Genet 11:e1004910. doi:10.1371/journal.pgen.1004910. 25569256 PMC4287439

[45] Halldorsson BV, Palsson G, Stefansson OA, Jonsson H, Hardarson MT, Eggertsson HP, Gunnarsson B, Oddsson A, Halldorsson GH, Zink F, Gudjonsson SA, Frigge ML, Thorleifsson G, Sigurdsson A, Stacey SN, Sulem P, Masson G, Helgason A, Gudbjartsson DF, Thorsteinsdottir U, Stefansson K. 2019. Human genetics: characterizing mutagenic effects of recombination through a sequence-level genetic map. Science 363:eaau1043. doi:10.1126/science.aau1043. 30679340

[46] Lynch M, Sung W, Morris K, Coffey N, Landry CR, Dopman EB, Dickinson WJ, Okamoto K, Kulkarni S, Hartl DL, Thomas WK. 2008. A genome-wide view of the spectrum of spontaneous mutations in yeast. Proc Natl Acad Sci USA 105:9272–9277. doi:10.1073/pnas.0803466105. 18583475 PMC2453693

[47] Wang L, Sun Y, Sun X, Yu L, Xue L, He Z, Huang J, Tian D, Hurst LD, Yang S. 2020. Repeat-induced point mutation in Neurospora crassa causes the highest known mutation rate and mutational burden of any cellular life. Genome Biol 21:142. doi:10.1186/s13059-020-02060-w. 32546205 PMC7296669

[48] Villalba de la Peña M, Summanen PAM, Liukkonen M, Kronholm I. 2022. Variation in spontaneous mutation rate and spectrum across the genome of Neurospora crassa. bioRxiv. doi:10.1101/gr.276992.122.

[49] Freitag M, Williams RL, Kothe GO, Selker EU. 2002. A cytosine methyltransferase homologue is essential for repeat-induced point mutation in Neurospora crassa. Proc Natl Acad Sci USA 99:8802–8807. doi:10.1073/pnas.132212899. 12072568 PMC124379

[50] Gladyshev E. 2017. Repeat-induced point mutation and other genome defense mechanisms in fungi. Microbiol Spectr 5. doi:10.1128/microbiolspec.FUNK-0042-2017. PMC5607778 28721856

[51] Gladyshev E, Kleckner N. 2016. Recombination-independent recognition of DNA homology for repeat-induced point mutation (RIP) is modulated by the underlying nucleotide sequence. PLoS Genet 12:e1006015. doi:10.1371/journal.pgen.1006015. 27148882 PMC4858203

[52] Selker E. 2002. Repeat-induced gene silencing in fungi, p 439–450. *In* Wu C-T, Dunlap J (ed), Homology effects. Academic, San Diego, CA. 10.1016/s0065-2660(02)46016-6 11931234

[53] Fudal I, Ross S, Brun H, Besnard A-L, Ermel M, Kuhn M-L, Balesdent M-H, Rouxel T. 2009. Repeat-induced point mutation (RIP) as an alternative mechanism of evolution toward virulence in Leptosphaeria maculans. Mol Plant Microbe Interact 22:932–941. doi:10.1094/MPMI-22-8-0932. 19589069

[54] van de Wouw AP, Cozijnsen AJ, Hane JK, Brunner PC, McDonald BA, Oliver RP, Howlett BJ. 2010. Evolution of linked avirulence effectors in Leptosphaeria maculans is affected by genomic environment and exposure to resistance genes in host plants. PLoS Pathog 6:e1001180. doi:10.1371/journal.ppat.1001180. 21079787 PMC2973834

[55] Hane JK, Williams AH, Taranto AP, Solomon PS, Oliver RP. 2015. Repeat-induced point mutation: a fungal-specific, endogenous mutagenesis process, p 55–68. *In* van den Berg MA, Maruthachalam K (ed), Genetic transformation systems in fungi, vol 2. Springer International Publishing, Cham, Switzerland.

[56] Goodwin SB, M’Barek SB, Dhillon B, Wittenberg AHJ, Crane CF, Hane JK, Foster AJ, Van der Lee TAJ, Grimwood J, Aerts A, Antoniw J, Bailey A, Bluhm B, Bowler J, Bristow J, van der Burgt A, Canto-Canché B, Churchill ACL, Conde-Ferràez L, Cools HJ, Coutinho PM, Csukai M, Dehal P, De Wit P, Donzelli B, van de Geest HC, van Ham RCHJ, Hammond-Kosack KE, Henrissat B, Kilian A, Kobayashi AK, Koopmann E, Kourmpetis Y, Kuzniar A, Lindquist E, Lombard V, Maliepaard C, Martins N, Mehrabi R, Nap JPH, Ponomarenko A, Rudd JJ, Salamov A, Schmutz J, Schouten HJ, Shapiro H, Stergiopoulos I, Torriani SFF, Tu H, de Vries RP, et al. 2011. Finished genome of the fungal wheat pathogen Mycosphaerella graminicola reveals dispensome structure, chromosome plasticity, and stealth pathogenesis. PLoS Genet 7:e1002070. doi:10.1371/journal.pgen.1002070. 21695235 PMC3111534

[57] Habig M, Quade J, Stukenbrock EH. 2017. Forward genetics approach reveals host genotype-dependent importance of accessory chromosomes in the fungal wheat pathogen Zymoseptoria tritici. mBio 8:e01919-17. doi:10.1128/mBio.01919-17. 29184021 PMC5705923

[58] Schotanus K, Soyer JL, Connolly LR, Grandaubert J, Happel P, Smith KM, Freitag M, Stukenbrock EH. 2015. Histone modifications rather than the novel regional centromeres of Zymoseptoria tritici distinguish core and accessory chromosomes. Epigenetics Chromatin 8:41. doi:10.1186/s13072-015-0033-5. 26430472 PMC4589918

[59] Habig M, Lorrain C, Feurtey A, Komluski J, Stukenbrock EH. 2021. Epigenetic modifications affect the rate of spontaneous mutations in a pathogenic fungus. Nat Commun 12:5869. doi:10.1038/s41467-021-26108-y. 34620872 PMC8497519

[60] Habig M, Kema GH, Stukenbrock EH. 2018. Meiotic drive of female-inherited supernumerary chromosomes in a pathogenic fungus. eLife 7:e40251. doi:10.7554/eLife.40251. 30543518 PMC6331196

[61] Morais D, Gélisse S, Laval V, Sache I, Suffert F. 2016. Inferring the origin of primary inoculum of Zymoseptoria tritici from differential adaptation of resident and immigrant populations to wheat cultivars. Eur J Plant Pathol 145:393–404. doi:10.1007/s10658-015-0853-y.

[62] Möller M, Schotanus K, Soyer JL, Haueisen J, Happ K, Stralucke M, Happel P, Smith KM, Connolly LR, Freitag M, Stukenbrock EH. 2019. Destabilization of chromosome structure by histone H3 lysine 27 methylation. PLoS Genet 15:e1008093. doi:10.1371/journal.pgen.1008093. 31009462 PMC6510446

[63] Grandaubert J, Bhattacharyya A, Stukenbrock EH. 2015. RNA-seq-based gene annotation and comparative genomics of four fungal grass pathogens in the genus Zymoseptoria identify novel orphan genes and species-specific invasions of transposable elements. G3 (Bethesda) 5:1323–1333. doi:10.1534/g3.115.017731. 25917918 PMC4502367

[64] Anderson CM, Chen SY, Dimon MT, Oke A, DeRisi JL, Fung JC. 2011. ReCombine: a suite of programs for detection and analysis of meiotic recombination in whole-genome datasets. PLoS One 6:e25509. doi:10.1371/journal.pone.0025509. 22046241 PMC3201961

[65] Bishop DK, Zickler D. 2004. Early decision: meiotic crossover interference prior to stable strand exchange and synapsis. Cell 117:9–15. doi:10.1016/s0092-8674(04)00297-1. 15066278

[66] McPeek MS, Speed TP. 1995. Modeling interference in genetic recombination. Genetics 139:1031–1044. doi:10.1093/genetics/139.2.1031. 7713406 PMC1206354

[67] Zhao H, Speed T, McPeek MS. 1995. Statistical analysis of crossover interference using the chi-square model. Genetics 139:1045–1056. doi:10.1093/genetics/139.2.1045. 7713407 PMC1206355

[68] Chen SY, Tsubouchi T, Rockmill B, Sandler JS, Richards DR, Vader G, Hochwagen A, Roeder GS, Fung JC. 2008. Global analysis of the meiotic crossover landscape. Dev Cell 15:401–415. doi:10.1016/j.devcel.2008.07.006. 18691940 PMC2628562

[69] Cummings WJ, Yabuki M, Ordinario EC, Bednarski DW, Quay S, Maizels N. 2007. Chromatin structure regulates gene conversion. PLoS Biol 5:e246. doi:10.1371/journal.pbio.0050246. 17880262 PMC1976632

[70] Bourque G, Burns KH, Gehring M, Gorbunova V, Seluanov A, Hammell M, Imbeault M, Izsvák Z, Levin HL, Macfarlan TS, Mager DL, Feschotte C. 2018. Ten things you should know about transposable elements. Genome Biol 19:199. doi:10.1186/s13059-018-1577-z. 30454069 PMC6240941

[71] Wells JN, Feschotte C. 2020. A field guide to eukaryotic transposable elements. Annu Rev Genet 54:539–561. doi:10.1146/annurev-genet-040620-022145. 32955944 PMC8293684

[72] Eickbush TH, Malik HS. 2007. Origins and evolution of retrotransposons, p 1111–1144. *In* Craig NL, Cragie R, Gellert M, Lambowitz AM (ed), Mobile DNA II. ASM Press, Washington, DC.

[73] Mazur AK, Gladyshev E. 2018. Partition of repeat-induced point mutations reveals structural aspects of homologous DNA-DNA pairing. Biophys J 115:605–615. doi:10.1016/j.bpj.2018.06.030. 30086830 PMC6104472

[74] Cambareri EB, Jensen BC, Schabtach E, Selker EU. 1989. Repeat-induced G-C to A-T mutations in Neurospora. Science 244:1571–1575. doi:10.1126/science.2544994. 2544994

[75] Martin SH, Davey JW, Salazar C, Jiggins CD. 2019. Recombination rate variation shapes barriers to introgression across butterfly genomes. PLoS Biol 17:e2006288. doi:10.1371/journal.pbio.2006288. 30730876 PMC6366726

[76] Wang S, Hassold T, Hunt P, White MA, Zickler D, Kleckner N, Zhang L. 2017. Inefficient crossover maturation underlies elevated aneuploidy in human female meiosis. Cell 168:977–989.e17. doi:10.1016/j.cell.2017.02.002. 28262352 PMC5408880

[77] Bhuiyan H, Schmekel K. 2004. Meiotic chromosome synapsis in yeast can occur without spo11-induced DNA double-strand breaks. Genetics 168:775–783. doi:10.1534/genetics.104.029660. 15514052 PMC1448848

[78] Storlazzi A, Tessé S, Gargano S, James F, Kleckner N, Zickler D. 2003. Meiotic double-strand breaks at the interface of chromosome movement, chromosome remodeling, and reductional division. Genes Dev 17:2675–2687. doi:10.1101/gad.275203. 14563680 PMC280617

[79] Tischfield SE, Keeney S. 2012. Scale matters: the spatial correlation of yeast meiotic DNA breaks with histone H3 trimethylation is driven largely by independent colocalization at promoters. Cell Cycle 11:1496–1503. doi:10.4161/cc.19733. 22433953 PMC3341227

[80] Fowler KR, Sasaki M, Milman N, Keeney S, Smith GR. 2014. Evolutionarily diverse determinants of meiotic DNA break and recombination landscapes across the genome. Genome Res 24:1650–1664. doi:10.1101/gr.172122.114. 25024163 PMC4199369

[81] Clouaire T, Legube G. 2015. DNA double strand break repair pathway choice: a chromatin based decision? Nucleus 6:107–113. doi:10.1080/19491034.2015.1010946. 25675367 PMC4615830

[82] Jeggo PA, Downs JA. 2014. Roles of chromatin remodellers in DNA double strand break repair. Exp Cell Res 329:69–77. doi:10.1016/j.yexcr.2014.09.023. 25278484

[83] Kalousi A, Soutoglou E. 2016. Nuclear compartmentalization of DNA repair. Curr Opin Genet Dev 37:148–157. doi:10.1016/j.gde.2016.05.013. 27266837

[84] Sung P, Klein H. 2006. Mechanism of homologous recombination: mediators and helicases take on regulatory functions. Nat Rev Mol Cell Biol 7:739–750. doi:10.1038/nrm2008. 16926856

[85] Alagoz M, Katsuki Y, Ogiwara H, Ogi T, Shibata A, Kakarougkas A, Jeggo P. 2015. SETDB1, HP1 and SUV39 promote repositioning of 53BP1 to extend resection during homologous recombination in G2 cells. Nucleic Acids Res 43:7931–7944. doi:10.1093/nar/gkv722. 26206670 PMC4652757

[86] Baldeyron C, Soria G, Roche D, Cook AJL, Almouzni G. 2011. HP1α recruitment to DNA damage by p150CAF-1 promotes homologous recombination repair. J Cell Biol 193:81–95. doi:10.1083/jcb.201101030. 21464229 PMC3082177

[87] Lee Y-H, Kuo C-Y, Stark JM, Shih H-M, Ann DK. 2013. HP1 promotes tumor suppressor BRCA1 functions during the DNA damage response. Nucleic Acids Res 41:5784–5798. doi:10.1093/nar/gkt231. 23589625 PMC3675466

[88] Soria G, Almouzni G. 2013. Differential contribution of HP1 proteins to DNA end resection and homology-directed repair. Cell Cycle 12:422–429. doi:10.4161/cc.23215. 23287531 PMC3587443

[89] Sun Y, Jiang X, Xu Y, Ayrapetov MK, Moreau LA, Whetstine JR, Price BD. 2009. Histone H3 methylation links DNA damage detection to activation of the tumour suppressor Tip60. Nat Cell Biol 11:1376–1382. doi:10.1038/ncb1982. 19783983 PMC2783526

[90] Ayrapetov MK, Gursoy-Yuzugullu O, Xu C, Xu Y, Price BD. 2014. DNA double-strand breaks promote methylation of histone H3 on lysine 9 and transient formation of repressive chromatin. Proc Natl Acad Sci USA 111:9169–9174. doi:10.1073/pnas.1403565111. 24927542 PMC4078803

[91] Lemaître C, Grabarz A, Tsouroula K, Andronov L, Furst A, Pankotai T, Heyer V, Rogier M, Attwood KM, Kessler P, Dellaire G, Klaholz B, Reina-San-Martin B, Soutoglou E. 2014. Nuclear position dictates DNA repair pathway choice. Genes Dev 28:2450–2463. doi:10.1101/gad.248369.114. 25366693 PMC4233239

[92] Schep R, Brinkman EK, Leemans C, Vergara X, van der Weide RH, Morris B, van Schaik T, Manzo SG, Peric-Hupkes D, van den Berg J, Beijersbergen RL, Medema RH, van Steensel B. 2021. Impact of chromatin context on Cas9-induced DNA double-strand break repair pathway balance. Mol Cell 81:2216–2230.e10. doi:10.1016/j.molcel.2021.03.032. 33848455 PMC8153251

[93] Tock AJ, Holland DM, Jiang W, Osman K, Sanchez-Moran E, Higgins JD, Edwards KJ, Uauy C, Franklin FCH, Henderson IR. 2021. Crossover-active regions of the wheat genome are distinguished by DMC1, the chromosome axis, H3K27me3, and signatures of adaptation. Genome Res 31:1614–1628. doi:10.1101/gr.273672.120. 34426514 PMC8415368

[94] van Wyk S, Wingfield BD, de Vos L, van der Merwe NA, Steenkamp ET. 2021. Genome-wide analyses of repeat-induced point mutations in the Ascomycota. Front Microbiol 11:622368. doi:10.3389/fmicb.2020.622368. 33597932 PMC7882544

[95] Coleman JJ, Rounsley SD, Rodriguez-Carres M, Kuo A, Wasmann CC, Grimwood J, Schmutz J, Taga M, White GJ, Zhou S, Schwartz DC, Freitag M, Ma L-J, Danchin EGJ, Henrissat B, Coutinho PM, Nelson DR, Straney D, Napoli CA, Barker BM, Gribskov M, Rep M, Kroken S, Molnar I, Rensing C, Kennell JC, Zamora J, Farman ML, Selker EU, Salamov A, Shapiro H, Pangilinan J, Lindquist E, Lamers C, Grigoriev IV, Geiser DM, Covert SF, Temporini E, VanEtten HD. 2009. The genome of Nectria haematococca: contribution of supernumerary chromosomes to gene expansion. PLoS Genet 5:e1000618. doi:10.1371/journal.pgen.1000618. 19714214 PMC2725324

[96] Cuomo CA, Güldener U, Xu J-R, Trail F, Turgeon BG, Di Pietro A, Walton JD, Ma L-J, Baker SE, Rep M, Adam G, Antoniw J, Baldwin T, Calvo S, Chang Y-L, Decaprio D, Gale LR, Gnerre S, Goswami RS, Hammond-Kosack K, Harris LJ, Hilburn K, Kennell JC, Kroken S, Magnuson JK, Mannhaupt G, Mauceli E, Mewes H-W, Mitterbauer R, Muehlbauer G, Münsterkötter M, Nelson D, O'donnell K, Ouellet T, Qi W, Quesneville H, Roncero MIG, Seong K-Y, Tetko IV, Urban M, Waalwijk C, Ward TJ, Yao J, Birren BW, Kistler HC. 2007. The Fusarium graminearum genome reveals a link between localized polymorphism and pathogen specialization. Science 317:1400–1402. doi:10.1126/science.1143708. 17823352

[97] Graïa F, Lespinet O, Rimbault B, Dequard-Chablat M, Coppin E, Picard M. 2001. Genome quality control: RIP (repeat-induced point mutation) comes to Podospora. Mol Microbiol 40:586–595. doi:10.1046/j.1365-2958.2001.02367.x. 11359565

[98] Idnurm A, Howlett BJ. 2003. Analysis of loss of pathogenicity mutants reveals that repeat-induced point mutations can occur in the Dothideomycete Leptosphaeria maculans. Fungal Genet Biol 39:31–37. doi:10.1016/S1087-1845(02)00588-1. 12742061

[99] Ikeda K, Nakayashiki H, Kataoka T, Tamba H, Hashimoto Y, Tosa Y, Mayama S. 2002. Repeat-induced point mutation (RIP) in Magnaporthe grisea: implications for its sexual cycle in the natural field context. Mol Microbiol 45:1355–1364. doi:10.1046/j.1365-2958.2002.03101.x. 12207702

[100] Pomraning KR, Connolly LR, Whalen JP, Smith KM, Freitag M. 2013. Repeat-induced point mutation, DNA methylation and heterochromatin in Gibberella zeae (anamorph: Fusarium graminearum), 93–109. *In* Brown DW, Proctor RH (ed), Fusarium genomics, molecular and cellular biology. Caister Academic Press, Norfolk, UK.

[101] van de Wouw AP, Elliott CE, Popa KM, Idnurm A. 2019. Analysis of repeat induced point (RIP) mutations in Leptosphaeria maculans indicates variability in the RIP process between fungal species. Genetics 211:89–104. doi:10.1534/genetics.118.301712. 30389803 PMC6325690

[102] Rouxel T, Grandaubert J, Hane JK, Hoede C, van de Wouw AP, Couloux A, Dominguez V, Anthouard V, Bally P, Bourras S, Cozijnsen AJ, Ciuffetti LM, Degrave A, Dilmaghani A, Duret L, Fudal I, Goodwin SB, Gout L, Glaser N, Linglin J, Kema GHJ, Lapalu N, Lawrence CB, May K, Meyer M, Ollivier B, Poulain J, Schoch CL, Simon A, Spatafora JW, Stachowiak A, Turgeon BG, Tyler BM, Vincent D, Weissenbach J, Amselem J, Quesneville H, Oliver RP, Wincker P, Balesdent MH, Howlett BJ. 2011. Effector diversification within compartments of the Leptosphaeria maculans genome affected by repeat-induced point mutations. Nat Commun 2:202. doi:10.1038/ncomms1189. 21326234 PMC3105345

[103] Frantzeskakis L, di Pietro A, Rep M, Schirawski J, Wu CH, Panstruga R. 2020. Rapid evolution in plant-microbe interactions: a molecular genomics perspective. New Phytol 225:1134–1142. doi:10.1111/nph.15966. 31134629

[104] Langmead B, Salzberg SL. 2012. Fast gapped-read alignment with Bowtie 2. Nat Methods 9:357–359. doi:10.1038/nmeth.1923. 22388286 PMC3322381

[105] McKenna A, Hanna M, Banks E, Sivachenko A, Cibulskis K, Kernytsky A, Garimella K, Altshuler D, Gabriel S, Daly M, DePristo MA. 2010. The Genome Analysis Toolkit: a MapReduce framework for analyzing next-generation DNA sequencing data. Genome Res 20:1297–1303. doi:10.1101/gr.107524.110. 20644199 PMC2928508

[106] Li H, Handsaker B, Wysoker A, Fennell T, Ruan J, Homer N, Marth G, Abecasis G, Durbin R, 1000 Genome Project Data Processing Subgroup. 2009. The Sequence Alignment/Map format and SAMtools. Bioinformatics 25:2078–2079. doi:10.1093/bioinformatics/btp352. 19505943 PMC2723002

[107] Quinlan AR. 2014. BEDTools: the Swiss-Army tool for genome feature analysis. Curr Protoc Bioinformatics 47:11.12.1–11.12.34. doi:10.1002/0471250953.bi1112s47. PMC4213956 25199790

[108] Danecek P, Auton A, Abecasis G, Albers CA, Banks E, DePristo MA, Handsaker RE, Lunter G, Marth GT, Sherry ST, McVean G, Durbin R, 1000 Genomes Project Analysis Group. 2011. The variant call format and VCFtools. Bioinformatics 27:2156–2158. doi:10.1093/bioinformatics/btr330. 21653522 PMC3137218

[109] Layer RM, Chiang C, Quinlan AR, Hall IM. 2014. LUMPY: a probabilistic framework for structural variant discovery. Genome Biol 15:R84. doi:10.1186/gb-2014-15-6-r84. 24970577 PMC4197822

[110] Chiang C, Layer RM, Faust GG, Lindberg MR, Rose DB, Garrison EP, Marth GT, Quinlan AR, Hall IM. 2015. SpeedSeq: ultra-fast personal genome analysis and interpretation. Nat Methods 12:966–968. doi:10.1038/nmeth.3505. 26258291 PMC4589466

[111] Lorrain C, Feurtey A, Möller M, Haueisen J, Stukenbrock E. 2021. Dynamics of transposable elements in recently diverged fungal pathogens: lineage-specific transposable element content and efficiency of genome defenses. G3 11:jkab068. doi:10.1093/g3journal/jkab068. 33724368 PMC8759822

[112] Wang X, Wang L. 2016. GMATA: an integrated software package for genome-scale SSR mining, marker development and viewing. Front Plant Sci 7:1350. doi:10.3389/fpls.2016.01350. 27679641 PMC5020087

[113] Heinz S, Benner C, Spann N, Bertolino E, Lin YC, Laslo P, Cheng JX, Murre C, Singh H, Glass CK. 2010. Simple combinations of lineage-determining transcription factors prime cis-regulatory elements required for macrophage and B cell identities. Mol Cell 38:576–589. doi:10.1016/j.molcel.2010.05.004. 20513432 PMC2898526

[114] Kema GHJ, Gohari AM, Aouini L, Gibriel HAY. 2018. Stress and sexual reproduction affect the dynamics of the wheat pathogen effector AvrStb6 and strobilurin resistance. Nat Genet 50:375–380. doi:10.1038/s41588-018-0052-9. 29434356

[115] Alexopoulos CJ, Mims CW, Blackwell M. 1996. Introductory Mycology. 4th ed, p 869. John Wiley & Sons, New York, USA.

