# Supplementary figures and images for: Repeat-Induced Point Mutation and Gene Conversion Coinciding with Heterochromatin Shape the Genome of a Plant-Pathogenic Fungus
Source: mBio. 2023 Apr 24;14(3):e03290-22. doi: 10.1128/mbio.03290-22 (PMC10294615; doi:10.1128/mbio.03290-22)

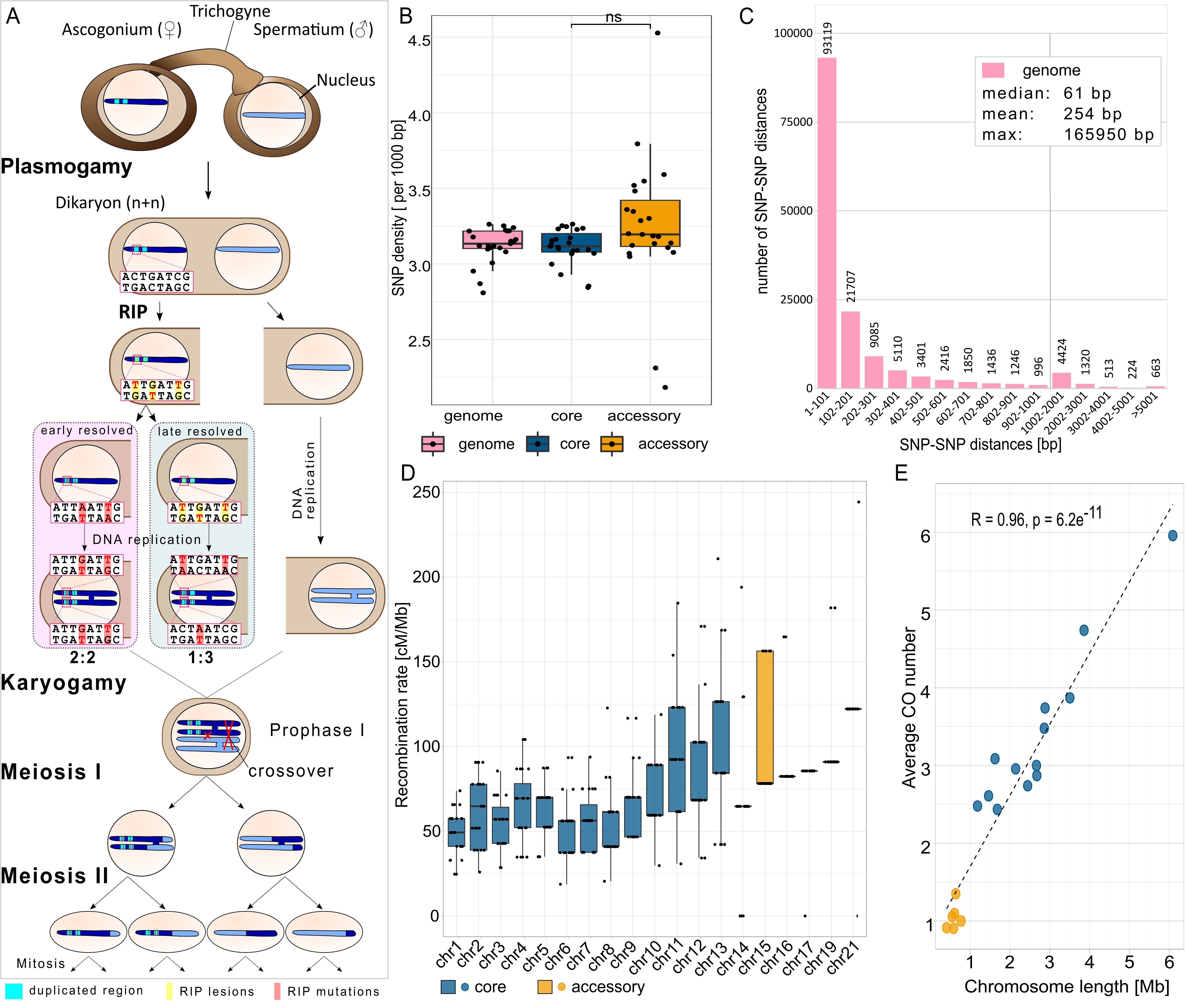

Supplement: FIG S1 [file mbio.03290-22-s0005.tif]

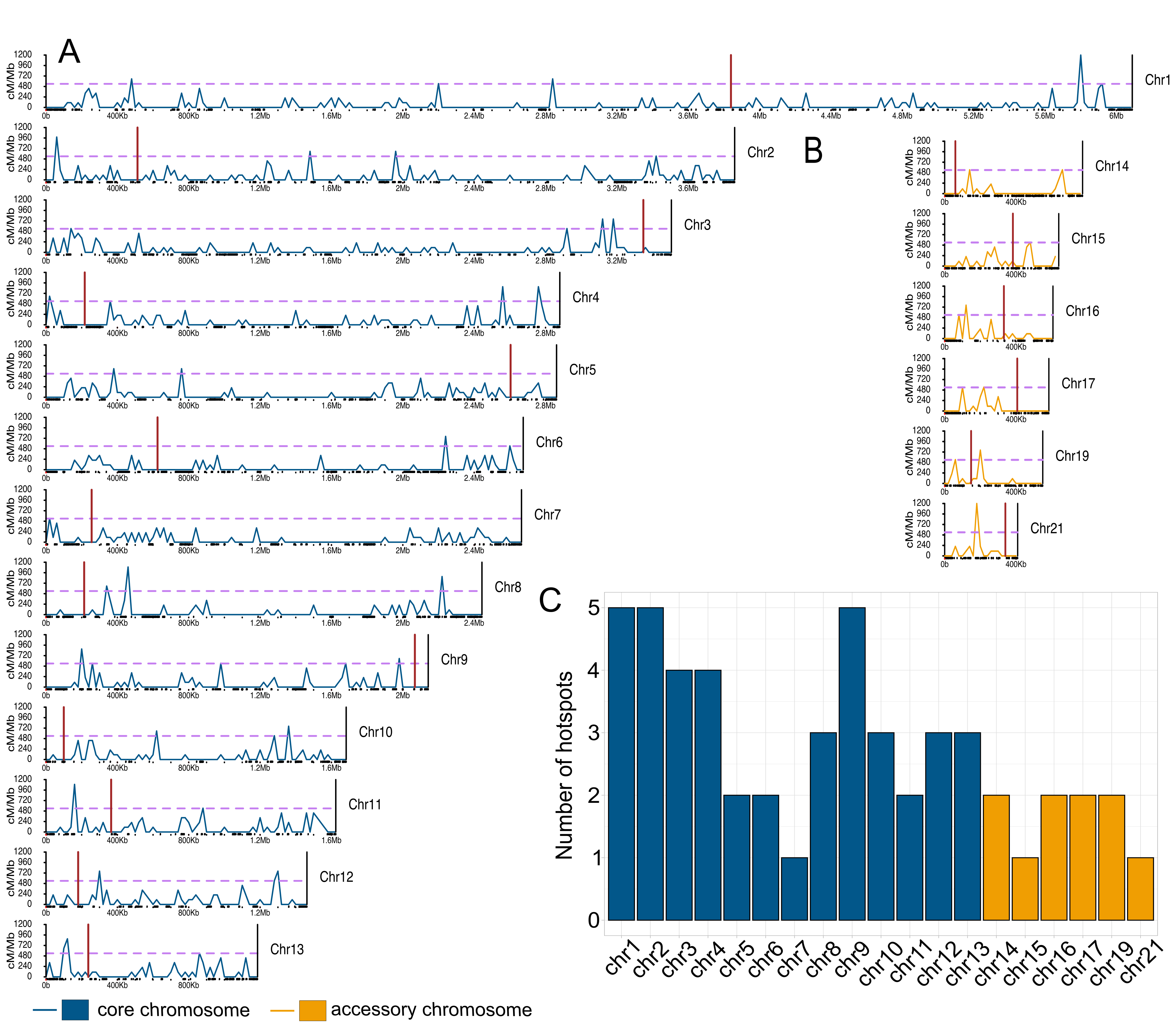

Supplement: FIG S2 [file mbio.03290-22-s0006.tif]

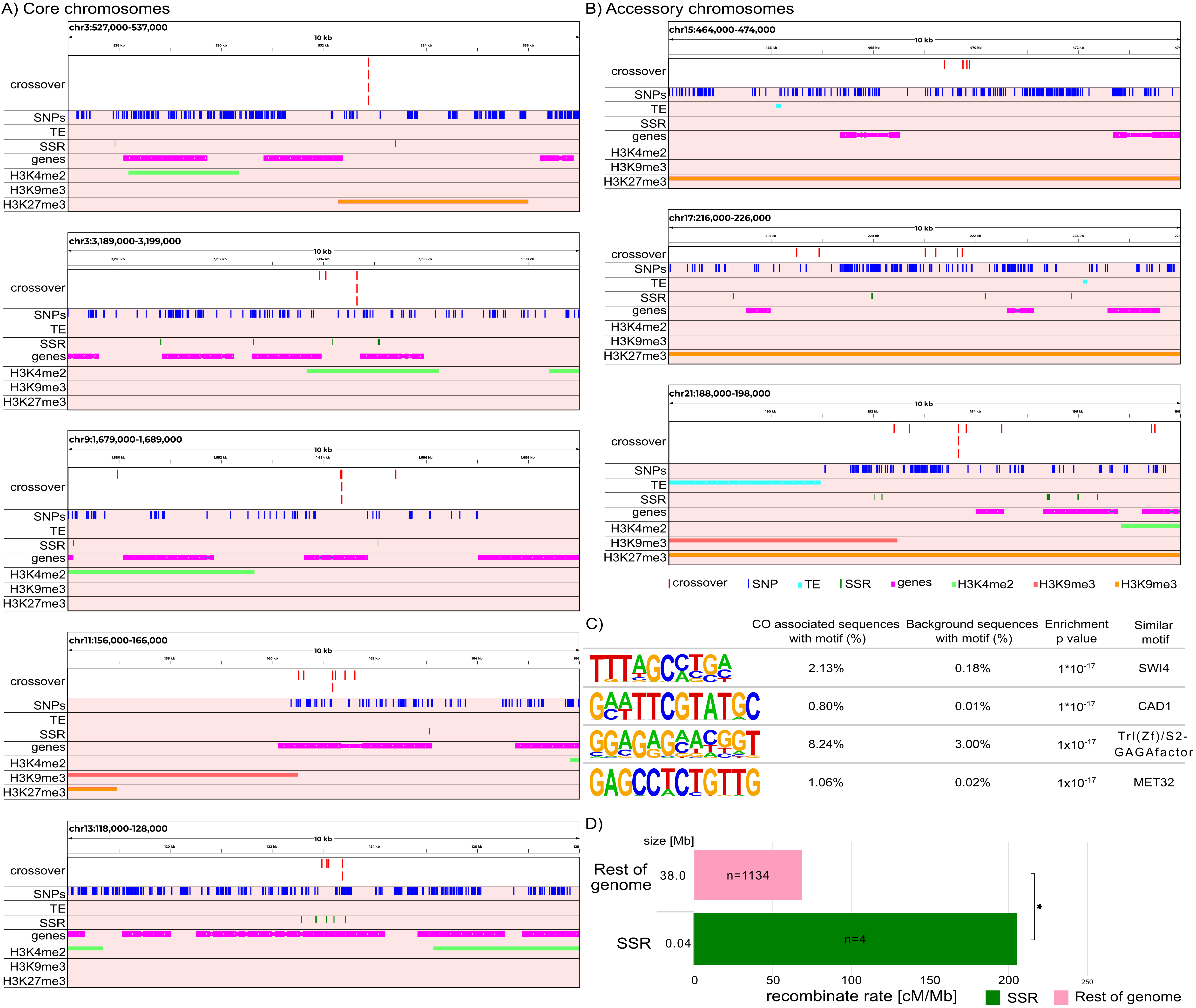

Supplement: FIG S3 [file mbio.03290-22-s0007.tif]

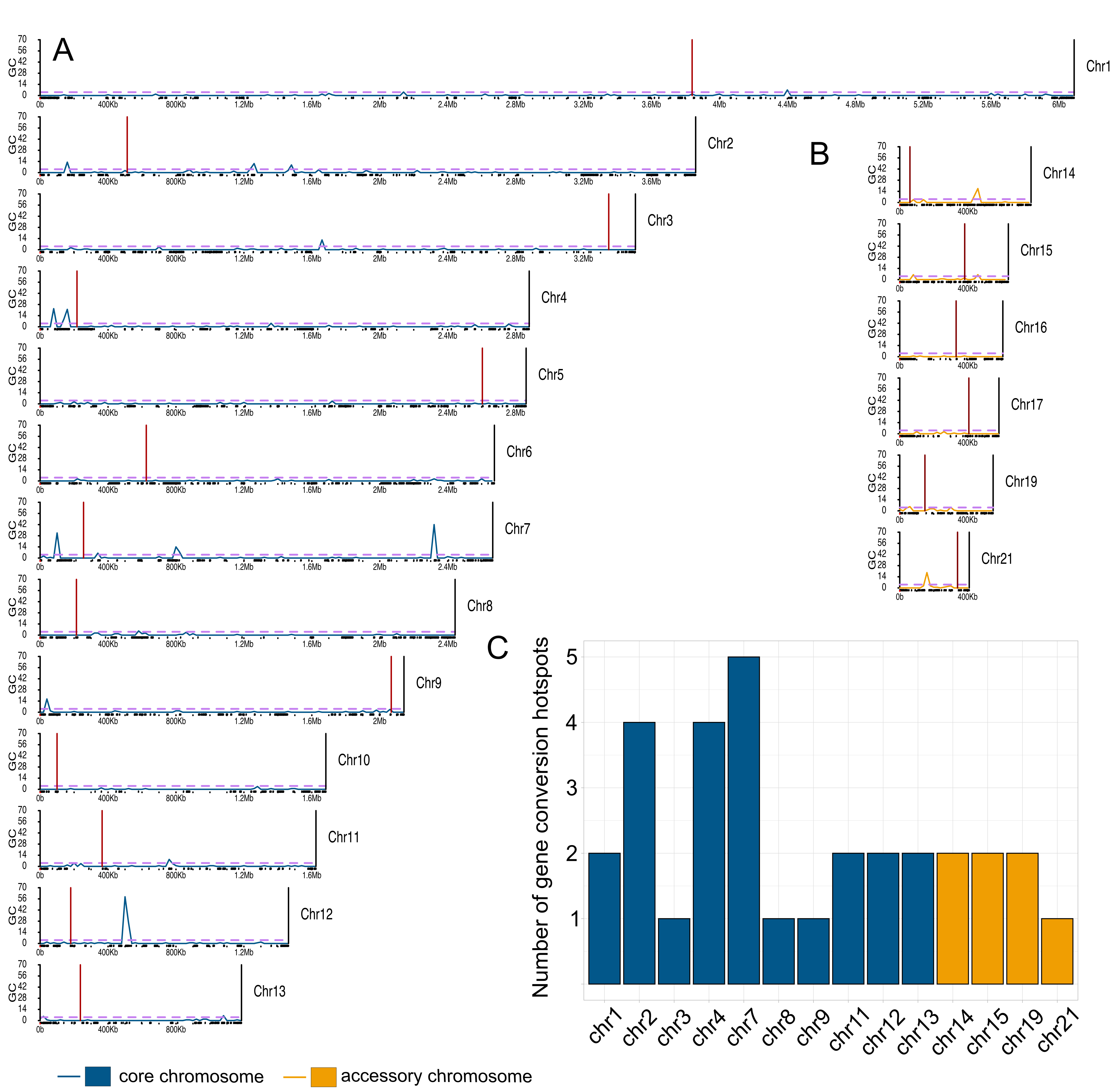

Supplement: FIG S4 [file mbio.03290-22-s0008.tif]

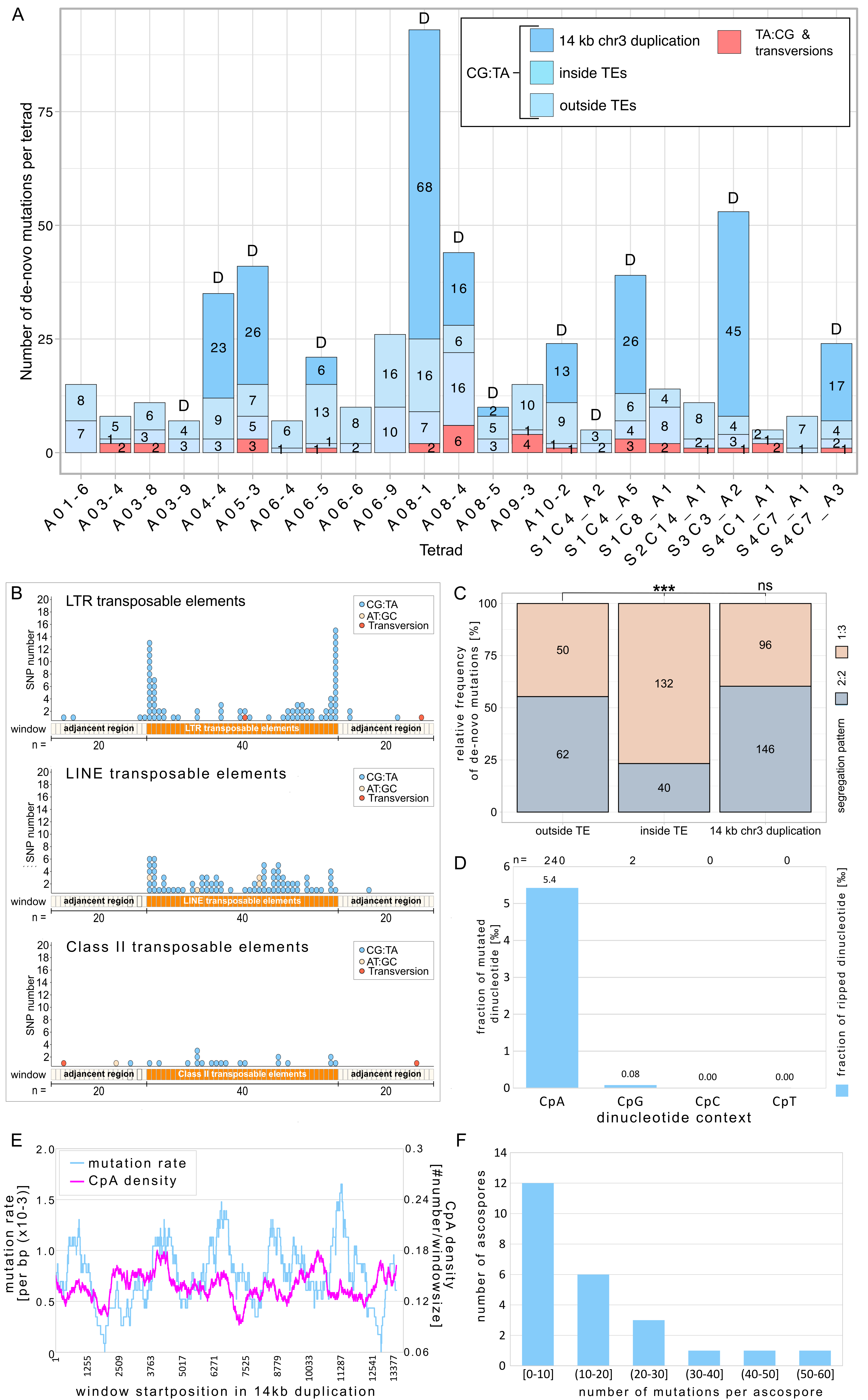

Supplement: FIG S5 [file mbio.03290-22-s0009.tif]

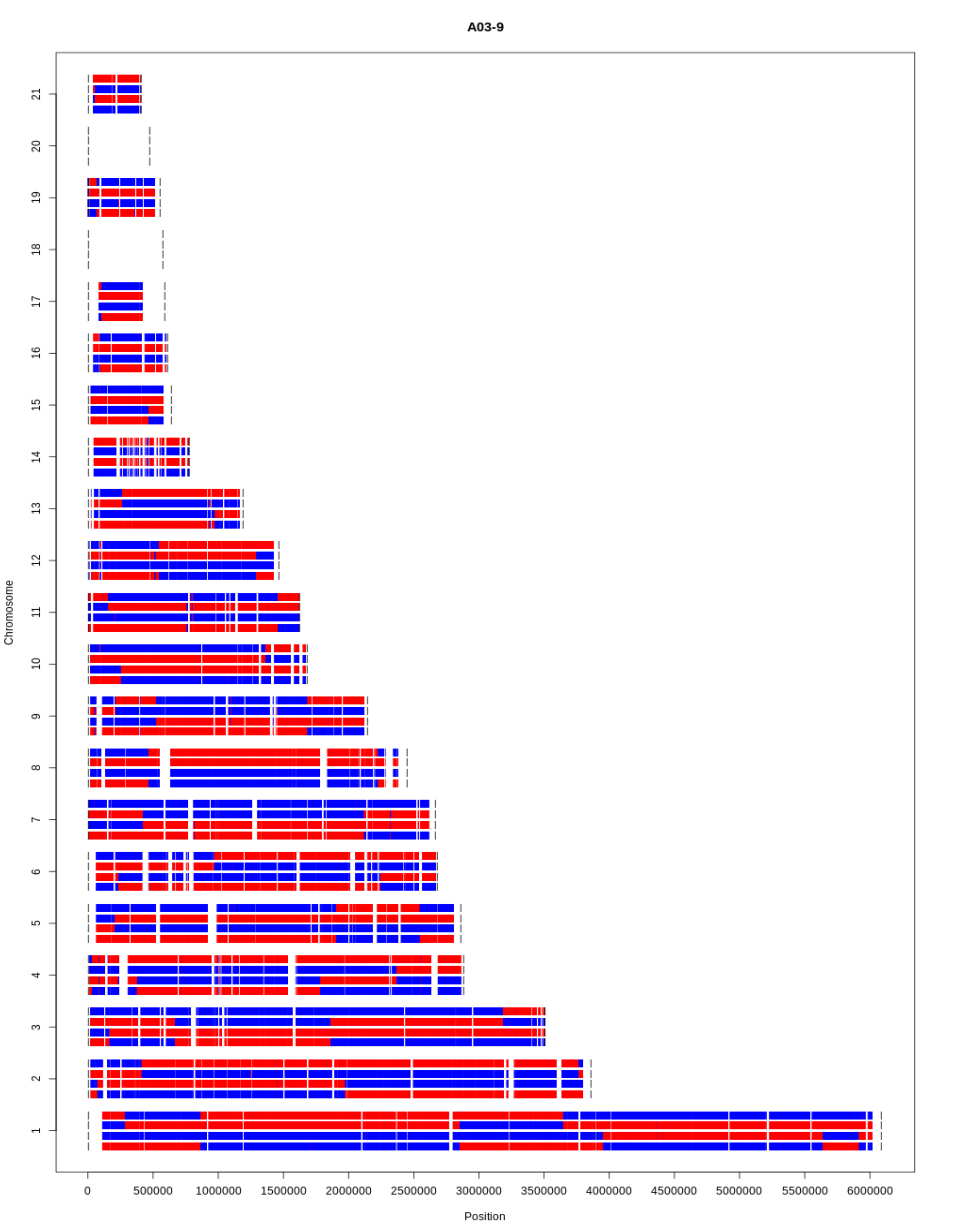

Supplement: FIG S6 [file mbio.03290-22-s0010.tif]
