# Supplementary material for: Repeat-Induced Point Mutation and Gene Conversion Coinciding with Heterochromatin Shape the Genome of a Plant-Pathogenic Fungus
Source: mBio. 2023 Apr 24;14(3):e03290-22. doi: 10.1128/mbio.03290-22 (PMC10294615; doi:10.1128/mbio.03290-22)
Supplement: TEXT S1 [file mbio.03290-22-s0001.pdf]

Supplementary Methods - Description of the bioinformatic procedures and tools and Sanger sequencing results.

A) Bioinformatic tools and procedures

#commands that have been used for bioinformatic analysis for mapping, SNP calling and creation of segregation files for the identification of crossover and gene conversion events# all lines that start with a single # are comments that explain the purpose of the command below the comment or explain the parameters/options that have been used for the command

1) checking the raw sequence quality (MultiQC v1.12)

```
# MultiQC is a tool that aggregate results from bioinformatics analyses across many samples into a single report
# this check is done to see the quality of fastq files from the sequencing facility and to decide which
# trimming parameters are used in the downstream processing
# '.' in the command marks all fastq files in the folder with the sequencing data

/folder_with_sequencing_data/ multiqc .

##########################################################################################
########################
```

2) trimming raw sequences (Trimmomatic version 0.36)

```
# PE -threads
# -phred33 quality score
java -jar Trimmomatic-0.36/trimmomatic-0.36.jar PE -phred33\
# forward input read file
 ${readdir}${file}_R1.fastq.gz\
# reverse input read file
 ${readdir}${file}_R2.fastq.gz\
# forward paired output file
 ${outdir}${file}_forward_paired.fq.gz\
# forward unpaired output file
 ${outdir}${file}_forward_unpaired.fq.gz\
# reverse paired output file
 ${outdir}${file}_reverse_paired.fq.gz\
# reverse unpaired output file
 ${outdir}${file}_reverse_unpaired.fq.gz\
```

```
# remove adapters (ILLUMINACLIP:TruSeq3-PE.fa:2:30:10)
# remove leading low quality or N bases (below quality 30) (LEADING:30)
# scan the read with a 4-base wide sliding window, cutting when the average quality per base drops
below 30 (SLIDINGWINDOW:4:30)
# drop the read if the average quality is below the specified level (AVGQUAL:30)
# drop reads below the 50 bases long (MINLEN:50)
 ILLUMINACLIP:TruSeq3-PE.fa:2:30:10 LEADING:30 SLIDINGWINDOW:4:30 AVGQUAL:30
MINLEN:50
####################################################################################
########################
```

3) checking the quality of trimmed sequences (MultiQC v1.12)

```
/folder_with_trimmed_data/ multiqc .

####################################################################################
########################
```

4) mapping the trimmed reads onto the reference IPO323 genome (bowtie2 version 2.3.4.1)

```
# -p number of threads
# --very-sensitive-local -setting for local alignment
# --rg-id ${FILE} -set the read group ID to ${FILE}
# --rg ${FILE} -add ${FILE} as a field on the read group header line of the output sequence alignment
map (sam) file
# -x reference genome
# -1 forward paired reads
# -2 reverse paired reads
# -S output sam file

bowtie2 -p 10 --very-sensitive-local --rg-id ${FILE} --rg SM:${FILE} -x IPO323.fa \
 -1 "${READDIR1}${FILE}_R1.fq.gz" -2 "${READDIR1}${FILE}_R2.fq.gz" \
 -U "${READDIR2}${FILE}_R1.fq.gz","${READDIR2}${FILE}_R2.fq.gz" \
 -S "${OUTDIR}${FILE}.sam"

####################################################################################
########################
```

5) converting (sequence alignment map) sam file to binary alignment map (bam) file (Picard v1.4.2)

```
java -jar picard.jar SortSam \
# setting the input file
I=${READDIR}${FILE}.sam \
```

# setting the output file
O=${OUTDIR}${FILE}.sorted.bam \
# sort the order of the output file
SORT_ORDER=coordinate

##
########################
6) quality check of the bam files generated in the step 5 (Qualimap v2.2.1)

#BAM QC reports information for the evaluation of the quality of all generated alignment data
-d,--data -file describing the input data. Format of the file is a 2-column tab-delimited table (column 1: sample name; column 2: path to BAM file (-r mode)
-outdir -output folder for report and raw data
-outformat -sets the format of the output report to PDF
-r,--run-bamqc -If this option is activated BAM QC process first will be run for each sample, then multi-sample analysis will be performed

qualimap multi-bamqc -d ${FILE} /path_to_file/${FILE} multibamqc.lst -outdir -outformat pdf -r

##
########################
7) SNP calling was performed with two variant callers samtools (version 1.7) and GATK (version 4.1.6.0). Common SNPs called by both
variant callers were extracted with bedtools intersect (described in the step 8).

7.1-SNP calling with samtools (version 1.7)
7.1a-creating file in pileup format for each spore from the sorted bam file generated in the step 5 and making a file that contains variants

#samtools mpileup – produces pileup textual format from an alignment
-E -recalculate BAQ (Phred-scaled probability of a read base being misaligned) on the fly, ignore existing BQ tags
-C50 -coefficient for downgrading mapping quality for reads containing excessive mismatches
-Q20 -minimum mapping quality for an alignment to be used
-q20 -minimum base quality for a base to be considered
-uf -reference genome file
samtools mpileup -E -C50 -Q20 -q20 -uf $IPO323.fa \
sorted bam file
${READDIR1}${FILE}.sorted.bam | \
bcftools call creates the raw binary variant call format (bcf) based on the bam file

```
# -vc -outputs variant sites only and use the original bcftools calling method
# -O u -O option determines the type of the output file (u-uncompressed bcf file)
# -o -determines the name of output file
bcftools call -vc -O u -o ${READDIR2}${FILE}.raw.bcf
```

7.1b-converting the binary variant files of each spore to the variant call format (vcf) file

```
bcftools view ${READDIR2}${FILE}.raw.bcf > ${READDIR3}${FILE}.raw.vcf
```

7.1c-filtering of the vcf files of each spore

```
# -i -includes the sites for which conditions below are true
# 'QUAL>90' -filtering sites based on QUAL (the Phred-scaled probability that the site has no variant)
# -q 0.9 -filters for alleles with the minimum allele frequency of 0.9
# --max-alleles 2 -filters only for biallelic SNPs
# --exclude-types indels -filters out indels from the final vcf file
bcftools view -i 'QUAL>90' -q 0.9 --max-alleles 2 --exclude-types indels\
${READDIR3}${FILE}.raw.vcf \
# -o -gives the name to the output file
-o ${OUTDIR}${FILE}.samtools.vcf
```

7.2 SNP calling with GATK (version 4.1.6.0)

7.2a-Adding and replacing read groups. A read group is a set of reads generated from a single run of sequencing instrument.
The read group tags are meant to enable grouping of alignment to account for biases due to things like e.g., the library preparation.

```
java -jar ~/tools/picard.jar AddOrReplaceReadGroups \
# I - setting the input bam file
    I=${READDIR}${FILE}.bam \
# O - setting the output bam file with added and replaced read groups
    O=${OUTDIR1}${FILE}.RG.bam \
# R - setting the reference genome file
    R=${READDIR2}IPO323.fa \
# RGID - setting the read-group ID
    RGID=1 \
# RGLB - setting the read-group library
    RGLB=lib1 \
# RGPL - setting the read-group platform
```

```
    RGPL=illumina \
# RGPU - setting the read-group platform unit (e.g. run barcode)
    RGPU=unit1 \
# RGSM - setting the read-group sample name
    RGSM=1
```

7.2b-Marking duplicates in the bam file of each spore generated in the step 7.2.a. Marking of duplicates is done to
remove PCR duplicates and to avoid risk of having over-representation in your sequence of areas preferentially amplified during PCR.

```
java -jar /home/jovan/tools/picard.jar MarkDuplicates \
# I - setting the input bam file
  I=${OUTDIR1}${FILE}.RG.bam \
# O - setting the output bam file with marked duplicates
  O=${OUTDIR}${FILE}.RG.MD.bam \
# M - setting files to write duplication metrics to
  M=${OUTDIR}/marked_dup_metrics.txt
```

7.2c-Indexing of the bam file with marked duplicates and added or replaced groups (output of the step 7.2b) with samtools option index (version 1.7).
Indexing of the bam fikes allows one to quickly extract alignments overlapping particular genomic regions.

```
samtools index ${OUTDIR}${FILE}.RG.MD.bam
```

7.2d-SNP calling with GATK HaplotypeCaller

```
# "-Xmx8G" -instructs Java to work with 8GB of memory (or RAM)
# --native-pair-hmm-threads -setting the number of threads that can be used

/home/jovan/tools/gatk-4.1.6.0/gatk --java-options "-Xmx8G" HaplotypeCaller --native-pair-hmm-threads 10 \
# R - setting the reference genome fasta file
-R ${READDIR2}IPO323.fa \
# I - setting the input bam file generated in the step 7.2c
-I ${OUTDIR}${FILE}.RG.MD.bam \
# O - setting the output vcf file
-O ${OUTDIR2}${FILE}.vcf
```

7.2e-filtering of the vcf files of each spore

```
# -i -includes the sites for which conditions below are true
# 'QUAL>90' -filtering sites based on QUAL (the Phred-scaled probability that the site has no variant)
# -q 0.9 -filters for alleles with the minimum allele frequency of 0.9
# --max-alleles 2 -filters only for biallelic SNPs
# --exclude-types indels -filters out indels from the final vcf file
bcftools view -i 'QUAL>90' -q 0.9 --max-alleles 2 --exclude-types indels\
${READDIR3}${FILE}.raw.vcf \
# -o -gives the name to the output file
-o ${OUTDIR}${FILE}.gatk.vcf
```

```
########################################################################################
#########################
```

8) creating the vcf file containing only SNPs called with GATK and samtools (bedtools v2.26.0)

```
# bedtools intersect performs feature intersection of -a vcf file generated with GATK and -b vcf file generated with samtools for each ascospore
bedtools intersect -a ${FILE}.gatk.vcf -b ${FILE}.samtools.vcf -header > ${FILE}.vcf
```

```
########################################################################################
#########################
```

9) merging vcf files of four spores into the single tetrad vcf file (tabix 1.9 and VCFtools v0.1.15)

9a) indexing of all vcf files

```
tabix -p vcf ${FILE}
```

9b) merging of the respective spores from a single tetrad

```
vcf-merge spore1.vcf.gz spore2.vcf.gz spore3.vcf.gz spore4.vcf.gz > tetrad.vcf.gz
```

```
########################################################################################
#########################
```

10) calculating and extracting regions with coverage > 5 for every spore (mosdepth version 0.2.6 and awk 1.3.3)

```
# this command will generate bed.gz file with coverages for each genome position;
# -t --threads: number of BAM decompression threads
# --fast-mode do not look at internal cigar operations or correct mate overlaps
10a) mosdepth -t 4 --fast-mode ${FILE} ${FILE}.bam

# filters the previously generated file for positions with coverage > 5:
```

in the output file from mosdepth the fourth column has coverage value for specific position
10b) awk '{if ($4 > 5) {print $1 "\t" $2 "\t" $3;} }' spore.coverage.bed.gz > spore.coverage6.bed.gz

##

11) filtering for regions that have coverage > 5 in all four spores of the tetrad (bedtools v2.26.0)

feature intersection to create bed file with coordinates of regions with the coverage >5 present in both spore1 and spore2
bgzip compresses the output bed file
11a) bedtools intersect -a spore1.coverage6.bed.gz -b spore2.coverage6.bed.gz | bgzip > spore12.coverage6.bed.gz

feature intersection of -a the bed file generated in the 11a step with -b bed file that has coordinates of regions with coverage >5 in spore3
11b) bedtools intersect -a spore12.coverage6.bed.gz -b spore3.coverage6.bed.gz | bgzip > spore123.coverage6.bed.gz

feature intersection of -a the bed file generated in the 11b step with -b bed file that has coordinates of regions with coverage >5 in spore4
output file is the bed file that contains regions with coverage >5 in all four spores of a tetrad
11c) bedtools intersect -a spore123.coverage6.bed.gz -b spore4.coverage6.bed.gz | bgzip > tetrad.coverage6.bed.gz

feature intersection of -a merged tetrad vcf file created in the step 9b with -b the bed file generated in the 11c step to get all SNPs
that are present only in the regions wirh coverage >5 in all four spores of a tetrad
-header writes the header of a -a vcf file to the output file
11d) bedtools intersect -a tetrad.vcf -b tetrad.coverage6.bed -header > tetrad.coverage6.vcf

##

12) filtering for regions without TEs (bedtools v2.26.0)

-a vcf file that contains all snps with coverage >= 6
-b bed file that contains coorsinates of all TEs in the genome of Z. tritici
-header saves the header of the vcf file in the output file
-v writes everything that is present in -a file but not in -b file to the output file (this option removes all SNPs that are present in the TEs)

```
bedtools intersect -a tetrad.coverage6.vcf -b TEs.bed -header -v > tetrad.coverage6-TE.vcf

###########################################################################################
##########################
```

13) converting merged tetrad file into the input segregation file for crossOver.py (awk 1.3.3, sed 4.4 and grep 3.1)

```
# removing all lines that start with '##' from the merged vcf file of a single tetrad generated in the step 12
grep -v '##' ${READDIR}tetrad.coverage6-TE.vcf | \
# substitutes all '.' characters with 0 because '.' indicates the same nucleotide as in the reference genome
sed -e "s/\./0/g" | \
# extracts column 1, 2, 10, 11, 12, 13 and inserts the third column with the name 'PlaceHolder' in which all lines
# have 'PlaceHolder' as a value. This column is necessary for CrossOver.py to work.
awk '{print $1 "\t" $2 "\tPlaceHolder\t" $10 "\t" $11 "\t" $12 "\t" $13 }' | \
# removes all 'chr' string from the file leaving only chromosome number in the first column
sed 's/chr//g' | \
# substitutes everything that is not equal to 0 with 1 in the fourth column (this column represents the segregation in the spore 1 of a tetrad)
awk 'BEGIN {OFS=FS="\t"} {if ($4!=0)sub($4,1); print }' | \
# substitutes everything that is not equal to 0 with 1 in the fifth column (this column represents the segregation in the spore 2 of a tetrad)
awk 'BEGIN {OFS=FS="\t"} {if ($5!=0)sub($5,1); print }' | \
# substitutes everything that is not equal to 0 with 1 in the sixth column (this column represents the segregation in the spore 3 of a tetrad)
awk 'BEGIN {OFS=FS="\t"} {if ($6!=0)sub($6,1); print }' | \
# substitutes everything that is not equal to 0 with 1 in the seventh column (this column represents the segregation in the spore 4 of a tetrad) and writes the segfile
awk 'BEGIN {OFS=FS="\t"} {if ($7!=0)sub($7,1); print }' > ${OUTDIR}tetrad.coverage6-TE.segfile.txt

#example of a segfile (0-IPO323 parent; 1-IPO94269 parent)
#the first 6 positions have 2:2 segregation; position 119117 has 3:1 segregation; positions 119120-119238 have 1:3 segregation; position 119308 has 0:4 segregation
```

#CHROM	POS	PlaceHolder	spore1	spore2	spore3	spore4
1	113241	PlaceHolder	0	0	1	1
1	115041	PlaceHolder	0	0	1	1
1	116317	PlaceHolder	0	0	1	1

1	116712	PlaceHolder	0	0	1	1
1	118362	PlaceHolder	0	0	1	1
1	119083	PlaceHolder	0	0	1	1
1	119117	PlaceHolder	0	1	1	1
1	119120	PlaceHolder	0	0	0	1
1	119184	PlaceHolder	0	0	0	1
1	119225	PlaceHolder	0	0	0	1
1	119238	PlaceHolder	0	0	0	1
1	119308	PlaceHolder	1	1	1	1

###

14) checking of the segregation ratios in the file generated in the step 13

We expect similar number of positions with 1:3 and 3:1 segregation. An over-representation of 1:3 segregation

tracts is a consequence of SNP not being called in the same position in some of the remaining spores and it leads to false positive gene conversion.

Therefore, the number of tracts with specific segregation (2:2, 1:3, 3:1 and 0:4) is counted prior to running the

analysis with CrossOver.py.

the segregation check is done by summing the values from spore1, spore2, spore3 and spore4 columns for each line

and counting the number of positions with specific segregation value (2 for 2:2, 1 for 1:3 tracts, etc.). In the example below,

positions 119120, 119184, 119225 and 119238 on chr1 have 1:3 segregation, and the position 119117 on chr1 3:1 segregation.

#CHROM	POS	PlaceHolder	spore1	spore2	spore3	spore4	segregation
1	113241	PlaceHolder	0	0	1	1	2
1	115041	PlaceHolder	0	0	1	1	2
1	116317	PlaceHolder	0	0	1	1	2
1	116712	PlaceHolder	0	0	1	1	2
1	118362	PlaceHolder	0	0	1	1	2
1	119083	PlaceHolder	0	0	1	1	2
1	119117	PlaceHolder	0	1	1	1	3
1	119120	PlaceHolder	0	0	0	1	1
1	119184	PlaceHolder	0	0	0	1	1
1	119225	PlaceHolder	0	0	0	1	1

1	119238	PlaceHolder	0	0	0	1	1
1	119308	PlaceHolder	1	1	1	1	4

15) If there is an over-representation of 1:3 tracts compared to 3:1 tracts the following steps are performed:

15.1) checking if 0 values (i.e. positions that are the same as in the reference genome) are also non-variants in files that were filtered for QUAL>30.

```
# Firstly, a separate segregation file was made for each spore with the following commands:
# if value in spore1 column, print #CHROM and POS column of tetrad segfile | insert chr to the beginning of each line
awk '{ if ($4==0) {print $1 "\t" $2};}' tetrad.segfile.txt | sed '/^/ s/^/chr/' > spore1.segfile.txt
# if value in spore2 column, print #CHROM and POS column of tetrad segfile | insert chr to the beginning of each line
awk '{ if ($5==0) {print $1 "\t" $2};}' tetrad.segfile.txt | sed '/^/ s/^/chr/' > spore2.segfile.txt
# if value in spore3 column, print #CHROM and POS column of tetrad segfile | insert chr to the beginning of each line
awk '{ if ($6==0) {print $1 "\t" $2};}' tetrad.segfile.txt | sed '/^/ s/^/chr/' > spore3.segfile.txt
# if value in spore4 column, print #CHROM and POS column of tetrad segfile | insert chr to the beginning of each line
awk '{ if ($7==0) {print $1 "\t" $2};}' tetrad.segfile.txt | sed '/^/ s/^/chr/' > spore4.segfile.txt

# the output files look as following:
# for spore1:
chr1	113241
chr1	115041
chr1	116317
chr1	116712
chr1	118362
chr1	119083
chr1	119117
chr1	119120
chr1	119184
chr1	119225
chr1	119238

# for spore2:
chr1	113241
chr1	115041
chr1	116317
```

chr1 116712
chr1 118362
chr1 119083
chr1 119120
chr1 119184
chr1 119225
chr1 119238

for spore3:
chr1 119120
chr1 119184
chr1 119225
chr1 119238

in this example, spore4 file would be empty because all values are equal to 1

15.2) comparing of files generated in the previous step with the vcf files of corresponding spores that were filtered out from the first dataset because they had QUAL<90 or were called by only one of the variant callers (this comparison was done separately for vcf files called with gatk and vcf files called with samtools):

grep -Fwf spore.segfile.txt spore.gatk.vcf > spore.gatk.vcf
grep -Fwf spore.segfile.txt spore.samtools.vcf > spore.samtools.vcf

grep prints lines that contain a match for one or more patterns and -Fwf option extracts variants from the vcf file if they are present in the spore segregation file:

-F
--fixed-strings

 # Interpret patterns as fixed strings, not regular expressions.

-w
--word-regexp

 # Select only those lines containing matches that form whole words.

-f file
--file=file

```
  #  Obtain patterns from file, one per line.

# below is the example of the output file of grep command for vcf file the spore3. The SNPs below were
filtered out from the primary dataset made in the step 14 because they were only called with one of
# the variant callers, but they satisfy all other filtering criteria and therefore it is likely that these SNPs
are true.

chr1    119120  .       T       G       1685.06 .
AC=2;AF=1;AN=2;DP=40;ExcessHet=3.0103;FS=0;MLEAC=2;MLEAF=1;MQ=42.72;QD=27.62;SOR=1.
04   GT:AD:DP:GQ:PL  1/1:0,38:38:99:1699,114,0
chr1    119184  .       C       T       1651.06 .
AC=2;AF=1;AN=2;DP=37;ExcessHet=3.0103;FS=0;MLEAC=2;MLEAF=1;MQ=42.61;QD=28.59;SOR=1.
121  GT:AD:DP:GQ:PL  1/1:0,37:37:99:1665,111,0
chr1    119225  .       A       G       1561.06 .
AC=2;AF=1;AN=2;DP=35;ExcessHet=3.0103;FS=0;MLEAC=2;MLEAF=1;MQ=42.59;QD=25.72;SOR=0.
869  GT:AD:DP:GQ:PL  1/1:0,35:35:99:1575,105,0
chr1    119238  .       C       A       759.06  .
AC=2;AF=1;AN=2;DP=19;ExcessHet=3.0103;FS=0;MLEAC=2;MLEAF=1;MQ=36.19;QD=33.74;SOR=0.
914  GT:AD:DP:GQ:PL  1/1:0,18:18:54:773,54,0

# output vcf files from both gatk and samtools are both appended to a single file
cat spore.gatk.vcf >> spore.gatksamtools.vcf
cat spore.samtools.vcf >> spore.gatksamtools.vcf
```

15.3) conversion of the vcf file obtained in the step 15.2 to segregation file

```
# prints the first two columns from a vcf file from 15.2 and prints the third column with the value 1
awk '{print $1 "\t" $2 "\t" "1"}' spore.gatksamtools.vcf | \
# removes the 'chr' from the first column
awk -F"\t" '{OFS="\t"; gsub("chr", "", $1); print }' | \
# removes all duplicated SNPs (duplicates can happen, e.g. if both variant callers called the same SNP
with QUAL<90, so this command leaves only single SNP)
awk '!seen[$1,$2,$4,$5]++' | \
# sorting the variants
sort --version-sort | \
# inserting header and writing the output file:
awk 'BEGIN{print "#CHROM" "\t" "POS" "\t" "spore"}1' $f > spore.gatksamtools.txt

# the output txt file from the output vcf file in step 15.2:
```

```
#CHROM  POS     spore3
1       119120  1
1       119184  1
1       119225  1
1       119238  1
```

15.4) extracting segregation of each spore from the merged segregation file generated in the step 14 for comparison with segregation files from the step 15.3:

```
awk '{print $1 "\t" $2 "\t" $4}' tetrad.segfile.txt > spore1.segfile.txt
awk '{print $1 "\t" $2 "\t" $5}' tetrad.segfile.txt > spore2.segfile.txt
awk '{print $1 "\t" $2 "\t" $6}' tetrad.segfile.txt > spore3.segfile.txt
awk '{print $1 "\t" $2 "\t" $7}' tetrad.segfile.txt > spore4.segfile.txt
```

output files for each spore look as following:

```
#file for spore 1:
#CHROM          POS     spore1
1       113241 0
1       115041 0
1       116317 0
1       116712 0
1       118362 0
1       119083 0
1       119117 0
1       119120 0
1       119184 0
1       119225 0
1       119238 0
1       119308 1

#file for spore 2:
#CHROM          POS     spore2
1       113241 0
1       115041 0
1       116317 0
1       116712 0
1       118362 0
1       119083 0
```

1 119117 1
1 119120 0
1 119184 0
1 119225 0
1 119238 0
1 119308 1

#file for spore 3:
#CHROM POS spore3
1 113241 1
1 115041 1
1 116317 1
1 116712 1
1 118362 1
1 119083 1
1 119117 1
1 119120 0
1 119184 0
1 119225 0
1 119238 0
1 119308 1

#file for spore 4:
#CHROM POS spore4
1 113241 1
1 115041 1
1 116317 1
1 116712 1
1 118362 1
1 119083 1
1 119117 1
1 119120 1
1 119184 1
1 119225 1
1 119238 1
1 119308 1

15.5) replacing false 0 values with value 1 in files generated in the step 15.4:
this is done with python script that is based on pandas package that allows comparison of dataframes

```
# importing necessary modules
import pandas as pd
import os
import numpy as np
import glob

# storing path to files generated in the step 15.4 as a variable
path = "/txt_files_from_step_15.4/*.txt"
# storing path to files generated in the step 15.3 as a variable
path2 = "/txt_files_from_step_15.3/"
# storing path for exporting files with substituted 0 values
path_out =
"/home/jovan/Desktop/practice4/tetrads_for_analysis_v3/columns_spores/spores_substituted_val1/"

# iteration
for fname in glob.glob(path):
    print(fname)
    basefname = os.path.basename(fname)
    print(basefname)
    # importing dataframes
    df1 = pd.read_csv(fname, delimiter="\t")
    df2 = pd.read_csv(path2 + basefname, delimiter="\t")

    # setting multi-indexing to allow matching based on the values from two columns
    df1 = df1.set_index(['#CHROM', 'POS'])
    df2 = df2.set_index(['#CHROM', 'POS'])

    # switching values on corresponding positions from df1 with values from df2
    df1.update(df2)
    df1.reset_index(inplace=True)

    # storing new dataframe into new folder
    df1.to_csv(path_out + basefname, index = False, float_format='%.0f', sep="\t")
```

If we take spore3 as an example in this script the df1 is:

#CHROM	POS	spore3
1	113241	1
1	115041	1

```
1	116317 1
1	116712 1
1	118362 1
1	119083 1
1	119117 1
1	119120 0
1	119184 0
1	119225 0
1	119238 0
1	119308 1
```

the df2 is:

```
#CHROM  POS     spore3
1	119120  1
1	119184  1
1	119225  1
1	119238  1
```

final segregation file of spore 3 is:

```
#CHROM	POS	spore3
1	113241 1
1	115041 1
1	116317 1
1	116712 1
1	118362 1
1	119083 1
1	119117 1
1	119120 1
1	119184 1
1	119225 1
1	119238 1
1	119308 1
```

15.6) joining individual spore segregation files from step 15.5 to a tetrad segregation file that will be input for CrossOver:

```
#importing necessary modules
import pandas as pd
import numpy as np
import os
from functools import reduce

path_in = "/path/to/single/spore/segfiles_step15.5/"
path_out = "/path/to/exporting/folder/"

# importing segfile of each spore as a separate dataframe
df1 = pd.read_csv(path_in + 'spore1.txt', delimiter="\t")
df2 = pd.read_csv(path_in + 'spore2.txt', delimiter="\t")
df3 = pd.read_csv(path_in + 'spore3.txt', delimiter="\t")
df4 = pd.read_csv(path_in + 'spore4.txt', delimiter="\t")


# compile the list of dataframes you want to merge
data_frames = [df1, df2, df3, df4]

# merging segfiles of each spore into single tetrad segfile
df_merged = reduce(lambda  left,right: pd.merge(left,right,on=['#CHROM', 'POS'],
                                    how='outer'), data_frames)

# inserting PlaceHolder column to the merged dataframe
df_merged['PlaceHolder'] = "PlaceHolder"

# ordering the columns of the merged dataframe
df_merged = [['#CHROM','POS','PlaceHolder','spore1','spore2','spore3','spore4']]

# exporting the merged tetrad segfile
df_merged.to_csv(path_out + 'tetrad.segfile.txt', sep='\t', index=False)

# the output file looks as following:
#CHROM       POS    PlaceHolder     spore1  spore2  spore3  spore4
1       113241 PlaceHolder     0       0       1       1
1       115041 PlaceHolder     0       0       1       1
1       116317 PlaceHolder     0       0       1       1
1       116712 PlaceHolder     0       0       1       1
1       118362 PlaceHolder     0       0       1       1
1       119083 PlaceHolder     0       0       1       1
```

1	119117	PlaceHolder	0	1	1	1
1	119120	PlaceHolder	0	0	1	1
1	119184	PlaceHolder	0	0	1	1
1	119225	PlaceHolder	0	0	1	1
1	119238	PlaceHolder	0	0	1	1
1	119308	PlaceHolder	1	1	1	1

the files generated in this step are used for the crossover and gene conversion identification with CrossOver.py.

#Comparison of numbers of 1:3 and 3:1 tracts for each tetrad is shown in the table below.

tetrad	Seg_1:3	Seg_3:1
A01-6	139	198
A03-4	137	124
A03-8	133	172
A03-9	144	132
A04-4	137	160
A05-3	131	160
A06-4	230	191
A06-5	162	244
A06-6	169	212
A06-9	154	169
A08-1	269	147
A08-4	144	207
A08-5	103	210
A09-3	161	141
A10-2	142	229
S1C4_A2	169	175
S1C4_A5	171	179
S1C8_A1	175	144
S2C14_A1	169	238
S3C3_A2	181	166
S4C1_A1	182	248
S4C7_A1	181	229
S4C7_A3	260	209

B) Verification of in silico detected meiotic mutations by Sanger Sequencing

To verify *in silico* detected meiotic mutations, we performed Sanger sequencing of 20 randomly selected mutations which were identified as de novo mutations for the absence in the parental strains and their presence in the ascospores. For 19 of the 20 of these mutations, the absence in the parental and the presence in the ascospores could be confirmed. One mutation could not be confirmed in the ascospore. The primers used and the results are summarized in Table 1.

Table 1: List and sequence of primer for PCR and Sanger sequencing and the number of SNPs that were confirmed/not confirmed.

Primer name	Direction	Coordinates	Sequence (5'-3')	Expected PCR product length (bp)	Number of SNPs for confirmation	Confirmed SNPs
oES4399	forward	1668351..1668370	TCGGGAGCTTGTTCCGAATC	823	1	0
oES4400	reverse	1669154..1669173	CCGAGAAGTTCCTTCCGCAT			
oES4409	forward	1671935..1671954	CCACTCTTCAAAGTCCCCGG	469	1	1
oES4410	reverse	1672384..1672403	CCTCCGTAATCGTCTGGCTC			
oES4411	forward	703864..703883	GTGAATGAGGGCAGACGGAC	533	1	1
oES4412	reverse	704377..704396	GAGATGGGCTTCTCGTTCCC			
oES4413	forward	779217..779236	CAGGCTCGATGAGGTGCTAG	548	2	2
oES4414	reverse	779745..779764	CACCGCCCCAAATAACGTCC			
oES4415	forward	2311522..2311541	CAAGGAGTCTAACAGGCGGG	770	1	1
oES4416	reverse	2312272..2312291	CCTCGTCCTTCTCCTTCTGC			
oES4419	forward	489832..489851	CACGGCAGGTAGAGGATACC	328	1	1
oES4420	reverse	490140..490159	GTAAGAGCGCTGACTGGTCC			
oES4423	forward	720521..720540	CTGGTGGTGCTCAAGGTCTC	583	1	1
oES4424	reverse	721084..721103	CATCCAGAGACTCCACCGTG			
oES4425	forward	1679865..1679884	CGGCCCTATAACCCTTGGAC	560	3	3
oES4426	reverse	1680405..1680424	GCTTGGAGGCGTATCAGAGG			
oES4447	forward	1669269..1669288	CGGTGAGTGACTGCCTGTAC	305	3	3
oES4448	reverse	1669554..1669573	ACAGGGGAGAGGAGATCAGG			
oES4449	forward	1680629..1680648	GATTCACCTCCCCACCTTCC	658	2	2
oES4450	reverse	1681267..1681286	CCCTCCAAGATGAGCGATCC			
oES4453	forward	1677596..1677615	CGAGAGATCCACCGTCGAAG	574	4	4
oES4454	reverse	1678150..1678169	GAAGAATGTGCATCGACCGC			
